# Chiral Hydroxylation at the Mononuclear Nonheme Fe(II) Center of 4-(*S*) Hydroxymandelate Synthase – A Structure-Activity Relationship Analysis

**DOI:** 10.1371/journal.pone.0068932

**Published:** 2013-07-23

**Authors:** Cristiana M. L. Di Giuro, Cornelia Konstantinovics, Uwe Rinner, Christina Nowikow, Erich Leitner, Grit D. Straganz

**Affiliations:** 1 Institute for Biotechnology and Biochemical Engineering, Graz University of Technology, Graz, Austria; 2 Institute of Organic Chemistry, University of Vienna, Vienna, Austria; 3 Institute of Analytical Chemistry and Food Chemistry, Graz University of Technology, Graz, Austria; University of Bologna & Italian Institute of Technology, Italy

## Abstract

(*S*)-Hydroxymandelate synthase (Hms) is a nonheme Fe(II) dependent dioxygenase that catalyzes the oxidation of 4-hydroxyphenylpyruvate to (*S*)-4-hydroxymandelate by molecular oxygen. In this work, the substrate promiscuity of Hms is characterized in order to assess its potential for the biosynthesis of chiral α-hydroxy acids. Enzyme kinetic analyses, the characterization of product spectra, quantitative structure activity relationship (QSAR) analyses and in silico docking studies are used to characterize the impact of substrate properties on particular steps of catalysis. Hms is found to accept a range of α-oxo acids, whereby the presence of an aromatic substituent is crucial for efficient substrate turnover. A hydrophobic substrate binding pocket is identified as the likely determinant of substrate specificity. Upon introduction of a steric barrier, which is suspected to obstruct the accommodation of the aromatic ring in the hydrophobic pocket during the final hydroxylation step, the racemization of product is obtained. A steady state kinetic analysis reveals that the turnover number of Hms strongly correlates with substrate hydrophobicity. The analysis of product spectra demonstrates high regioselectivity of oxygenation and a strong coupling efficiency of C-C bond cleavage and subsequent hydroxylation for the tested substrates. Based on these findings the structural basis of enantioselectivity and enzymatic activity is discussed.

## Introduction

**Figure 1:**
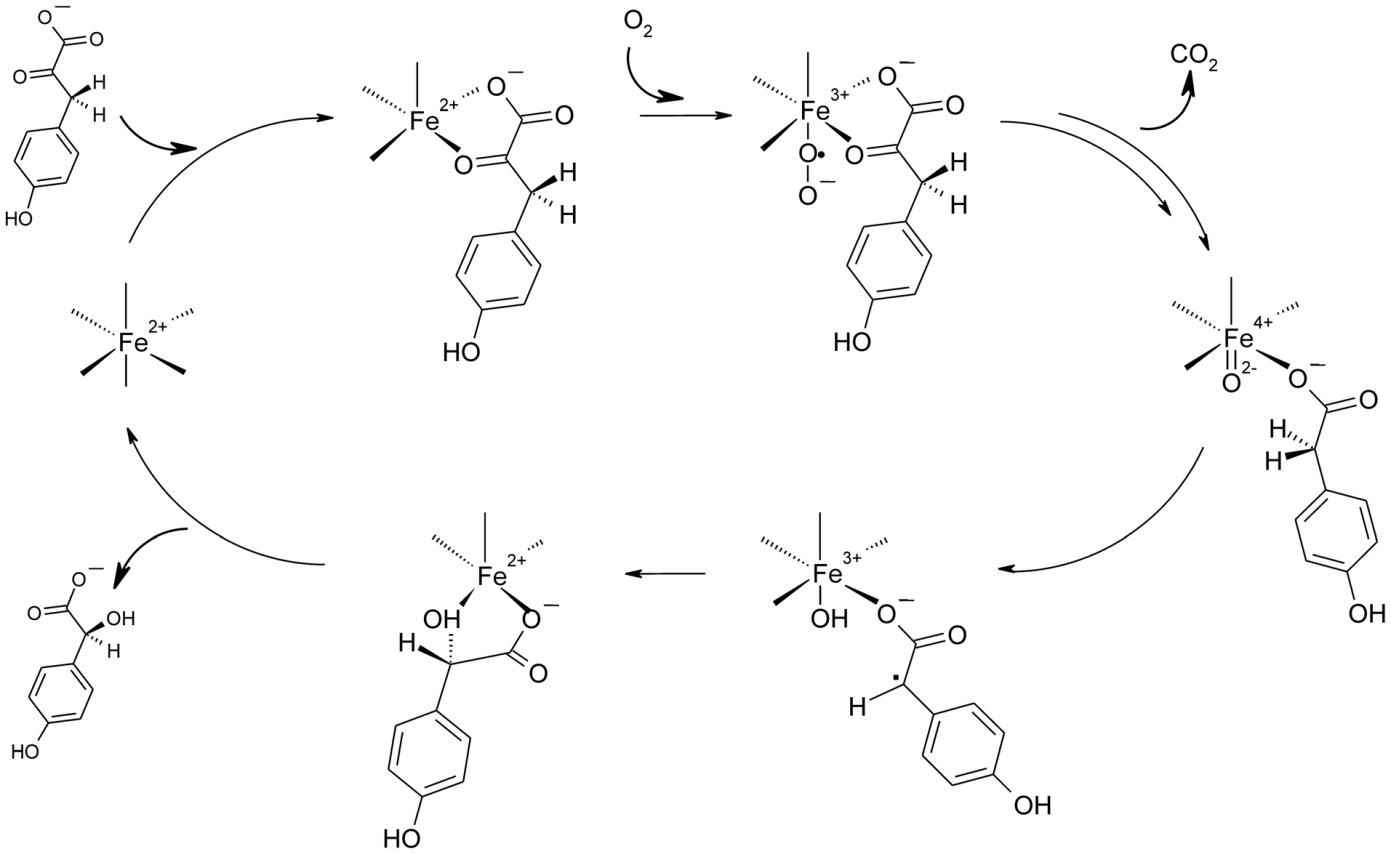
Principle proposed reaction mechanism of Hms [5].

Chiral aromatic and aliphatic α-hydroxy acids are important building blocks in the pharmaceutical chemistry [1] and their production via enzymatic conversions has been the target of intense research in the past few years. In search of green chemical routes towards α-hydroxy acid production *p*-hydroxymandelate synthase (Hms) is a potentially interesting target enzyme. The O_2_ dependent nonheme Fe(II) dioxygenase (MNHE) performs the chemically challenging chiral oxidation of the general metabolite *p*-hydroxyphenylpyruvate (HPP) to (*S*)-*p*-hydroxymandelate. Hms has first been reported as an enzyme of the biosynthetic pathway of the calcium dependent antibiotic CDA in *Amycolatopsis orientalis*, where it catalyzes the first step in the synthesis of the building block *p*-hydroxyphenylglycine [2], [3]. An analogous pathway has also been reported *for Streptomyces coelicolor* A3(2) [4]. The mechanism of Hms follows that of α-oxo acid dependent dioxygenases. After decarboxylation of HPP in an O_2_ dependent reaction Hms yields a high valent Fe(IV)-oxo intermediate. This highly reactive species abstracts an α-hydrogen from the 4-hydroxyphenylacetate intermediate and hydroxylates it in a rebound mechanism, thus yielding the product (Figure 1) [5].

Hms shows high structural similarity to *p*-hydroxyphenylpyruvate dioxygenase (Hppd), an enzyme that is found in the tyrosine metabolism, where it converts HPP to homogentisate. Hppd shares the initial reaction steps with Hms, however, in this enzyme the Fe(IV)-oxo intermediate performs an electrophilic attack of the aromatic ring. A subsequent rearrangement via an NIH shift then yields the product homogentisate. Conserved amino acid residues have been identified via mutational and bioinformatic analyses that allow to distinguish proteins with Hms activity from those with Hppd activity based on their primary sequence and the introduction of Hppd-type activity into an Hms via directed evolution approaches has successfully been demonstrated [6], [7]. Active site residues of Hppd that are crucial regarding substrate binding and the correct positioning of the reaction intermediate for oxygenation and for the subsequent NIH shift have been identified [8], [9]. Taken together, these studies highlight the importance of the interaction of protein and substrate structure for an efficient, site directed oxygenation reaction. The structure of Hms from *A. orientalis* has been solved [10] and based on kinetic analyses, which have revealed a lack of kinetic isotope effects on the turnover number, it has recently been concluded that product release is the rate limiting step regarding the conversion of the native substrate, HPP, by *A. orientalis* Hms [11].

Quantitative structure-activity relationship analysis (QSAR) has been used previously in order to gain insights into the mechanisms of mononuclear nonheme iron dioxygenases. Reaction kinetics of a series of biomimetic Fe(II) complexes show that the O_2_ dependent turnover of the substituted aromatic oxo acid benzoylformate positively correlates with the Hammett parameter. Consequently, a nucleophilic attack of superoxide at the α-oxo functionality has been proposed [12], [13]. A study of diketone dioxygenase Dke1 has shown that electronic effects have a strong impact on the primary rate of O_2_ reduction at enzymatic mononuclear nonheme iron centers [14] and also on the subsequent substrate cleavage pattern [15]. Electronic effects also determine phenylpyruvic acid cleavage. It has been demonstrated that the molecule can be cleaved by O_2_ via two distinct principle pathways, decarboxylation or C2-C3 bond cleavage [16]. MCD and Resonance Raman spectroscopic studies have revealed that the cleavage pathway depends on the electronic properties of the substrate coordinated Fe(II) center via the substrate’s protonation state: The 2-His 1-carboxylate motif, which is prototypical for MNHEs, stabilizes the ligation of the aromatic α-oxo acid in the monoanionic form, while an atypical enzymatic 3-His Fe(II) center stabilizes the dianion and this determines whether decarboxylation or C2-C3 bond cleavage occurs upon oxidation by O_2_ [17].

In this study for the first time the substrate specificity of an Hms towards a range of aliphatic and aromatic α-oxo acids has been investigated in order to gain insights into the structural basis of chiral hydroxylation by Hms. The substrate’s structural features that are required to induce dioxygen reduction at the metal center of Hms have been characterized. The impact of electronic and steric properties of the substrate structure on particular steps of subsequent catalysis has been assessed, with a focus on (i) the turnover number, (ii) the state of substrate ionization at the metal center, which is mirrored by distinct regioselectivities of oxygenative carbon-carbon bond cleavage and results in distinct product spectra [17], (iii) a putative decoupling of decarboxylation and hydroxylation and (iv) the regio- and enantioselectivity of hydroxylation. Therefore, Hms from *Streptomyces coelicolor* A3(2) has been functionally expressed and biochemically characterized. Its activity towards a range of aliphatic and aromatic α-oxo acids, some of them chemically synthesized for that purpose, has been characterized and subjected to quantitative structure activity relationship analysis (QSAR). Results show a strong preference of Hms for aromatic substrates and a quantifiable impact of the substrate structure on the enzyme’s turnover number. Also, a remarkable impact of the substrate structure on the reaction’s enantioselectivity is found, while no influence on the regioselectivity of oxygenation is observed. These findings are correlated with computational analyses to shed light on the structural features of Hms that impact the particular steps of catalysis. The study gives new insights into the catalytic potential of Hms for the biosynthesis of α-hydroxy acids.

## Materials and Methods

### 1. Chemicals and chemical synthesis

**Figure 2:**
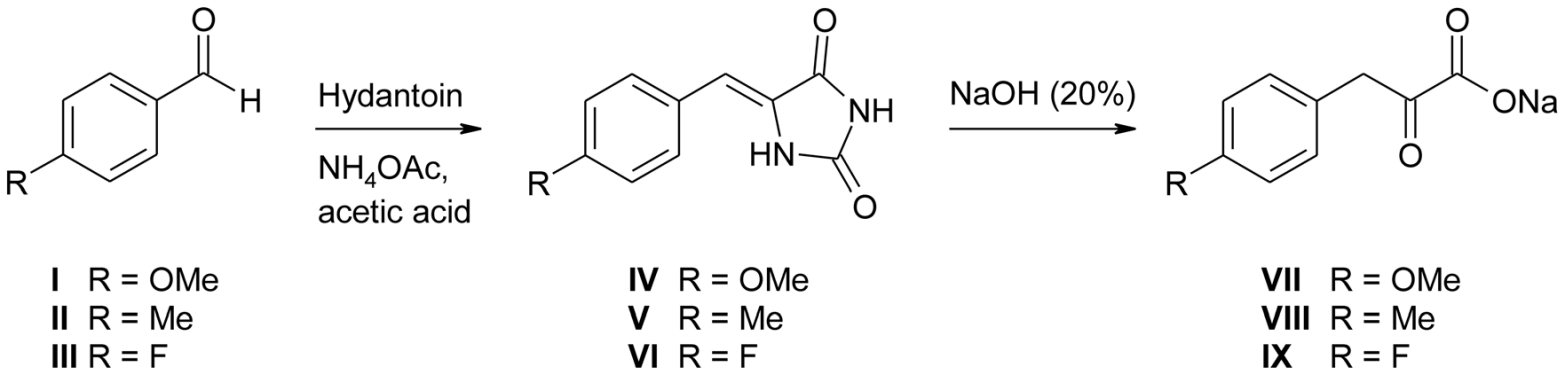
Preparation of α-oxo acids VII, VIII, and IX from the corresponding aromatic aldehydes.

*p*-Nitrophenylpyruvic acid was from Frinton laboratories Inc. (Vineland, NJ, U. S. A.), *p*-fluoromandelic acid was from Fluorochem (Hadfield, U. K.), (*R*)-*p*-methylmandelic acid was purchased at ABCR GmbH (Karlsruhe, Germany), while racemic *p*-methylmandelic acid was from Apollo Scientific (Bredbury, U. K). (*R*)-*p* methylmandelic acid was purchased at PARAGOS (Herdecke, Germany). Sodium phenylpyruvate and 3-methyl-2-oxobutyric acid were obtained from Fluka (St. Louis, MO, U. S. A.) and *p*-hydroxyphenylpyruvic acid (HPP), *p*-methoxymandelic acid, 2-oxooctanoid acid, 2-oxobutyric acid, 2-oxovaleric acid, *p*-nitrophenylpyruvic acid, 2-oxo-4-methylthiobutyric acid, 2-oxobutyric acid and all other chemicals and HPLC standards were purchased from Sigma Aldrich (St. Louis, MO, U. S. A.) at highest available purities. The synthesis of *p*-methoxypyruvic acid (**VII**), *p*-methylphenylpyruvic acid (**VIII**), and *p*-fluorophenylpyruvic acid (**IX**) is outlined in Figure 2. In summary, the corresponding aromatic aldehydes (**I**-**III**) were condensed with hydantoin to allow the isolation of the corresponding heterocyclic intermediates **IV**-**VI**. Hydrolysis of these intermediary formed imidazolidines under basic conditions then delivered the desired α-keto acids **VII**-**IX** [18]. Full experimental accounts are provided in the Supporting Information of this article (Protocol S1).

### 2. Strain and media

*Streptomyces coelicolor* A3(2) was obtained from `Deutsche Sammlung von Mikroorganismen und Zellkulturen GmbH' ( DSMZ ID no. 40783). The strain was kept on *Streptomyces* ‘medium 65’ plates, according to the manufacturer’s recommendation. *E. coli* BL21(DE3) from Stratagene (La Jolla, CA, U. S. A.) and *E. coli* Rosetta 2 from Merck KGaA (Darmstadt, Germany) were used as expression hosts for Hms. *E. coli* cells were kept on agar plates of Luria broth medium supplemented with 50 mg · mL^−1^ kanamycin and with 0.05 mg · L^−1^ of ampicillin and 0.06 mg · L^−1^ of chloramphenicol for *E.coli* BL21 (DE3) and Rosetta 2 cells respectively.

### 3. Cloning of p-hydroxymandelate synthase Hms from *Streptomyces coelicolor* A3(2)

Enzymes for molecular biological experiments were purchased from MBI Fermentas GMBH (St. Leon-Rot, Germany) if not stated otherwise. Genomic DNA from *S. coelicolor* A3(2) was obtained from cells that had been grown in ‘medium 65’ liquid broth for 24 hours at 30°C, harvested and washed with ice cold 50 mM KH_2_PO_4_/K_2_HPO_4_ buffer (pH 7.5) using the ‘DNA purification Wizard’ of Promega (Madison, WI, U. S. A.) according to the manufacturer’s procedure. Cells were broken in the presence of 20 mg · mL^−1^ lysozyme.

Primers to amplify Hms were designed based on the annotated sequence information of *S. coelicolor* A3(2) genomic DNA [19]. (a.) For the cloning of Hms bearing a C-terminal streptavidin affinity (strep) tag (*Strep*-tag II; IBA GmbH, Goettingen, Germany) the forward primer 5’ CTA TAC ATA TGC TCC CTC CTT TCC CCT TCC TTC 3’ was engineered with an *Nde*I site (underlined) which overlapped the initiation codon of *hms*. The reverse primer 5’ ATA GAG GAT CCT TA**T TTT TCG AAC TGC GGG TGG CTC CAA GCG CT**T CGG CCG GCC ACT TCC CG 3’, downstream of the stop codon, was designed with a *Bam*HI site (underlined) and a streptavidin affinity tag (in bold). Amplification was performed in a total volume of 50 µL with 5 Units µl^−1^ of Taq polymerase, 50 ng of chromosomal DNA from *S*. *coelicolor* A3(2) as a template, 50 pmol of each primer, 1 µL of dNTPs (1 mM each), 5 µL of Taq polymerase buffer, 4% DMSO, applying 30 cycles at 94°C (30 s), 68°C (30 s) and 72°C (60 s). Note that high temperatures and DMSO concentrations had to be applied and a range of polymerases with proof reading activity did not yield amplification product from chromosomal DNA, presumably due to the high content of GC (>70%) of the *S. coelicolor* A3(2) genome. (b.) In order to obtain N-terminally strep-tagged Hms the following primers were used: the forward primer was 5’ATG GTA GAA GAC AAG CGC GTG CTC CCT CCT TTC CCC TTC 3’, the reverse primer was 5’ATG GTA GAA GAC AAT 3’. The amplification was performed in a final volume of 50 µl with 2.5 Units µl^−1^ of Pfu polymerase, using 100 ng of the *hms* bearing vector pKYB1 (vide supra) resulting from above, 25 pmol of each primer 1 µL of dNTPs (200 µM each) 5 µL of Pfu polymerase buffer, 4% DMSO and applying 30 cycles at 95°C (90 s), 65°C (30 s) and 72°C (90 s).

DNA fragments from (a.) were analysed and purified using agarose gel electrophoresis, extracted using the QIAquick PCR purification kit protocol (Quiagen, Hilden, Germany) and cloned into the *Nde*I/*Bam*HI restriction sites of the expression vector pKYB1 (Stratagene, La Jolla, CA, U. S. A.), thus allowing inducible expression of recombinant Hms from the strong T7 promoter. The DNA fragments from (b.) were cut with *Bsa*I and ligated into a *Bsa*I cut p*ASK*-IBA 7 plus vector (IBA GmbH) using standard procedures. All constructs were subjected to dideoxy-sequencing of the entire *hms* gene in both directions, to verify that the clones bore the desired mutation and that no errors had been introduced into the sequence during amplification via PCR.

### 4. Protein production and purification

*E. coli* BL21(DE3) cells bearing the C-terminally strep-tagged Hms expression construct were grown in a culture medium that had been optimized previously for the production of an Fe(II) oxygenase using a previously described procedure [20]. *E. coli* Rosetta 2 cells bearing the N-terminally strep-tagged Hms expression construct were grown in a culture medium with 0.05 mg · L^−1^ ampicillin and 0.06 mg · L^−1^ cloramphenicol as a selection marker. When the main culture showed an OD_600_ of 0.8, expression was induced by adding 200 µg · L^−1^ anhydrotetracycline. Cells were grown for 24 hours at 18°C and then harvested by centrifugation at 6000 rpm at 4°C for 20 min. Cell pellets were resuspended in ∼30 ml of Tris/HCl buffer (20mM, pH 7.5) and the ice-cold suspension was subjected to 2 cycles of cell disruption in the French Press at 8500 kPa and subsequent centrifugation at 70,000 g for 2 hours. Affinity purification was carried out at 4°C using a 5 ml *Strep*–Tactin column (IBA, Goettingen, Germany) according to the manufacturer’s protocol as described previously [21]. Purified protein was then concentrated 20-fold using Vivaspin centrifugation concentration tubes (Sartorius AG, Goettingen, Germany) and subjected to three cycles of buffer exchange via size exclusion chromatography using a Nap-25^TM^ column (GE Healthcare, Chalfont St. Giles, U. K.). Protein purity was assessed by SDS PAGE according to standard procedures [22].

#### Gel Filtration

The size of Hms was estimated via gel filtration using ∼ 1 mg of purified protein with a Superdex 200 gel filtration column (25 ml; GE Healthcare) under standard conditions, using 20 mM MES buffer, pH 7.5, and 0.15 M NaCl as an eluent. A flow rate of 0.4 mL · min^−1^ was applied. Calibration was performed using a Gel Filtration Standard Kit (Bio-Rad, Hercules, CA, U. S. A.).

### 5. Protein concentration and iron content

Protein concentrations were determined via UV/Vis spectroscopy by measuring the absorbance of an appropriately diluted protein solution (K_2_HPO_4_/KH_2_PO_4_, 50 mM, pH 7.5) at 280 nm and by calculating the concentration with a theoretical ε, which had been determined based on the protein sequence [23], [24]. To test for the presence of iron in the enzyme preparation, the FereneS reaction was used: Fe(II) concentrations were determined spectrophotometrically by monitoring the formation of the coloured Fe(II)-FereneS chelate complex as described previously [25], [26] and apparent first order rate constants of iron detachment were determined as described previously [27]. All measurements were performed in Tris/HCl buffer, 20 mM and pH 7.5. The spectrophotometric method was validated using ICP-MS.

### 6. Enzyme activity and substrate binding assays

To determine enzymatic activity, O_2_ consumption rates were continuously monitored using a micro-optode O_2_ sensor (Microtox TX3-AOT, Presens GmbH, Regensburg, Germany). Assays were generally performed at 25°C in air saturated 20 mM Tris/HCl buffer, pH 7.5, with enzyme concentrations of ∼ 20 µM. Enzyme concentrations are generally expressed as the concentration of metal loaded enzymatic active sites. To start the reaction, substrate aliquots were added from 5 mM stock solutions of the respective substrate to the desired end concentration in a total volume of 500 µL. Initial rates were determined by linear regression analysis using the initial, linear part of the O_2_ depletion curve. O_2_ consumption rates from blanks that contained substrate in the absence of enzyme were subtracted as necessary. Specific activities were obtained by relating measured rates of O_2_ depletion to the Fe(II) loaded enzymatic active sites in the assay. Note that no additional Fe(II) was added to the assays, as we had found previously that oxidation of free Fe(II) interferes with the determination of enzymatic O_2_ consumption rates [28]. The correlation of O_2_ consumption with product formation was confirmed via HPLC-MS analysis. Spectrophotometric substrate binding assays were performed by anaerobic titration using a sealed cuvette as described previously [13], whereby all transfers into the cuvette were performed in an anaerobic glove box and an O_2_ concentration <10 µM in the reaction mixture prior to and after spectrophotometric measurements was generally confirmed via the micro-optode sensor.

### 7. Chromatographic methods

#### Product analysis via HPLC

Cleavage products of PP by Hms were determined via HPLC-MS on a chiral Chiralpak AD-RH column (25 cm; Diacel, Illkirch Cedex, France) using a system from Agilent (1200 series; Santa Clara, CA, U. S. A.) with a variable wavelength detector, which operated at an analytical wavelength of 210 nm, and an MS detector (model G1956B), which was used in negative mode and with APCI ionization and. A mixture of 88% water, 12% acetonitril (ACN) and 0.1% TFA was used as an eluent. Analyses were performed at 30°C column temperature using a flow rate of 0.5 mL · min^−1^. The elution of substrate and products was monitored at 210 nm. The identity of reaction products from enzymatic conversions was confirmed via HPLC-MS.

#### Product analysis via enantiomeric GC-MS

Contrary to the other investigated mandelate analogues, the unsubstituted mandelic acid enantiomers could not be separated via HPLC-MS. Consequently, chiral GC-MS chromatography was applied. The two enantiomeric mandelic acid compounds were separated on a BGB 174 chiral column (30 m, 0.25 mm inner diameter, 0.25 µm film thickness; BGB Analytik AG, Boeckten, Switzerland) after esterfication according to the procedure described by Kezic et al. [29]. For the derivatization procedure 1 mL of the aqueous sample was mixed with 1 mL 1.25 M HCl in isopropanol in a 4 mL glass vial with PTFE lined screw cap and heated for 30 min to 100°C. After cooling to room temperature 1 mL heptane was added and shaken for 1 min. Aliquots of 50 µl of the organic layer were transferred into 2 mL autosampler vials with a 250 µL microinsert and closed with PTFE lined crimp caps.

Samples were measured on an Agilent 7890 GC equipped with a combi PAL autosampler (CTC Analytics AG, Zwingen, Switzerland) coupled to a Agilent 5975 mass selective detector (Agilent Technologies Inc, Santa Clara, CA, U. S. A.). For analysis 1 µL of the extract was injected in a split/splitless injector operated in the splitless mode at an injection port temperature of 220°C. The split valve was opened 1 min after the injection. Helium was used as the carrier gas in the constant flow mode with a linear velocity of 24 cm s^−1^. A temperature program with a starting temperature of 100°C (1 min) and ramped with 4°C min^−1^ to 180°C was used. For the identification and quantification of the substrates a SIM/scan method was applied. SIM traces (107, 106, 79 and 77 m/z) were used for the quantification while the scan trace was used to monitor possible interferences with a scan range of 40–300 amu. For a positive identification the following criteria were used: (i) Retention time match with a pure reference compound, (ii) target ion, (iii) qualifier ion with a deviation of ±20% of the abundance ratio of target and qualifier ion in comparison with the pure reference substance. Under the given chromatographic conditions a base line separation of the two methylmandelate compounds was achieved. The retention times of the (*R*)-mandelic acid and *S*)-mandelic acid isopropyl esters were determined with 17.3 min and 18.3 min, respectively. Several attempts to establish a method for the determination of *p*-hydroxymandelic acid enantiopurity were not fruitful.

### 8. Circular dichroism spectroscopic studies

CD spectroscopic measurements for Hms were performed. The far-UV CD spectra were recorded with a Jasco J-715 spectropolarimeter at 25°C using 0.02 and 0.10 cm path length cylindrical cells. The instrument parameters were operated with a step resolution of 0.2 nm and a scan speed of 50 nm · min^−1^. Response was 1 s and bandwidth was 1 nm. Enzymes were prepared in 20 mM Tris/HCl buffer, pH 7.5, at a protein concentration of 3.0 mg · mL^−1^. Spectra obtained in the wavelength range of 300–190 nm were averaged and corrected by a blank spectrum lacking the enzyme before converting the signal into mean residue ellipticity. Data were elaborated using the software DICHROWEB [30], [31].

### 9. Quantitative structure activity relationship and in silico docking analyses

In order to investigate the impact of substrate and product hydrophobicity, average log *P* values were calculated using the online Virtual Computational Chemistry Laboratory VCCLAB (2005), at http://www.vcclab.org [32]. From the substrates’ and products’ log *P* values, log D values were derived, which consider dissociation of the acidic compound, based on the formula log *D*  =  log *P* + log {1/[1+10?(p*H*-p*K*_a_)]}. p*K*_a_ values were calculated using Advanced Chemistry Development Software V11.02 (© 1994–2013 ACD/Labs, Toronto, Canada). log *D* values were then correlated with log *k*_cat_. Additionally, log *P* values for the substituents of the substrates’ oxo ethanoate moiety as well as for the products’ hydroxy-ethanoate core structure were correlated with log *k*_cat_.

Electronic substituent effects on the rate determining step were investigated by correlating log *k*_cat_ with a range of parameters that were deduced from different models [33]: Beside the parameter originally defined by Hammett for para-substituted aromatic systems (σ), further tabulated parameters that describe the stabilization of positive (σ_p_^+^) or negative (σ_p_^−^) charge or the stabilization of a radical species (σ_p_^•^) during the transition state were employed. Furthermore a correlation of log *k*_cat_ with σ^*^, which accounts for a substituent’s aliphatic inductive effect, was performed. For structures where the α-oxo acid moiety is not directly fused to the aromatic ring, this parameter may be more applicable, as it probes the inductive effect of the respective substituent at C-3 of the pyruvate moiety, such as a phenyl-ring in the case of PP [33]. The substrate ligands’ highest occupied molecular orbital energies (εHOMO) and the atomic volumes of the respective substituents were obtained from semi-empirical calculations using the software Spartan (Wavefunction Inc., Irvine, CA, U. S. A.).

For in silico docking and structural analyses models of Hms from *S. coelicolor* A3(2) and from *A. orientalis* were built based on the enzymes’ primary sequences using the SWISS-MODEL server [34], [35] and were validated via PROCHECK [36]. The *A. orientalis* Hms model was generated, in order to account for residues of the protein that are missing in the crystal structure. The Hms crystal structure (pdb: 2RV5), which was used as a template, has Co(II) bound in the active site as a redox inactive Fe(II) mimic [10]. In our models the Co(II) ion was replaced by Fe(II) to allow the docking program the application of the respective integrated iron(II) force field parameters. AutoDock Vina was used for molecular docking and scoring [37]. All rotatable bonds of the ligand were considered in the docking process in order to identify its possible binding conformation with Hms. The number of grid points in xyz was set to 60, 60, 60, the spacing value was equivalent to 0.375 Å and the metal atom of Hms was regarded as grid center. Other parameters were default values implemented by the program. The AutoDock tools suite was used to add hydrogens, to assign partial Gasteiger charges and to merge nonpolar hydrogens and their charges with the parent carbon atom to the protein model and ligand.

In order to identify putative channels for substrate access and product release in the structures of *A. orientalis* and *S. coelicolor* Hms, the molecular surface of the structural models was calculated using the YASARA Structure suite version 12.11.20 (YASARA Biosciences) [38]. By visual inspection in each model a channel was detected that spanned the protein and connected the active site with the protein surface. However, in the *S. coelicolor* model one channel entrance was apparently blocked by one amino acid (Tyr359), while in the *A. orientalis* counterpart both channel ends were blocked by one amino acid each, namely Tyr339 and Phe188. In order to assess, whether low energy forms of the protein in an open channel conformation are feasible, an ensemble of protein models was generated, where the respective residues adopted distinct orientations according to the penultimate rotamer library [39], which is integrated into the Swiss-PdbViewer software [40]. Energy minimized structures were then generated using the YASARA suite [38]. A nonperiodic simulation cell around the protein monomer and extended by 5 Å in each direction was defined and YASARA’s p*K*_a_ values at pH 7.5 were assigned [41]. The system was energy minimized using a steepest descent minimization to remove conformational stress, followed by a simulated annealing minimization until convergence (<0.05 kJ mol^−1^ per 200 steps). Integration time steps were set to 1.33 and 4 fs for intra- and intermolecular forces. The potential energy of each protein structure was calculated and compared to the energy minimized model that had the original topomer conformation in order to determine the impact of the conformational change on the structural energy. Resulting low energy models were analyzed by calculating their molecular surfaces and by subsequently inspecting them for a putative open channel.

## Results

### 1. Isolation and biochemical characterization of Hms from *Streptomyces coelicolor* A3(2)

Hms from *Streptomyces coelicolor* A3(2) was functionally expressed in *E.coli* and purified by standard procedures (see Protocol S2 and Figure S1 for details). The protein preparation contained C-terminally strep-tagged Hms in its monomeric form according to gel filtration and showed a secondary structure composition of 16% alpha helix, 33% beta sheet and 20% random coil based on CD spectroscopic analysis. C-terminally tagged Hms eluted as a monomer on a gel filtration column (Figure S2). The N-terminally tagged protein, by contrast, was not properly folded, formed aggregates and showed no measurable enzymatic activity (Table S2) and was therefore not further included in this study. Properties of the enzyme are summarized in Table S1. The Fe(II) content of C-terminally tagged Hms was ∼40% and it was not significantly increased upon subsequent addition of ascorbic acid (5 mM) as a reducing agent, ruling out the presence of significant amounts of Fe(III) in the protein preparation. Iron detachment rates were measured at 21°C and gave an apparent first order rate constant of 0.0006 s^−1^, which is comparable with reported values for Hppd [26]. The enzymatic activity of C-terminally tagged Hms, further on termed Hms, towards its native substrate was determined under standard conditions (Tris/HCl buffer, pH 7.5, 20 mM, 25°C) at air saturation and a specific activity of 4.5 s^−1^ was determined.

### 2. Substrate spectrum of Hms

In order to assess the substrate spectrum of Hms, O_2_ consumption rates of the enzyme (0.1 mM enzyme active sites) in the presence of a range of commercially available α-oxo acids (5 mM) were recorded. Aliphatic substrates generally showed activities that were 10^3^ fold to >10^5^ fold lower than towards the native substrate HPP. Results are detailed in Table 1. Of all aliphatic substrates tested, only 2-oxo-4-methylthiobutyric acid (0.007 s^−1^), 2-oxooctanoic acid (0.001 s^−1^) and 3-methyl-2-oxobutyric acid (0.0006 s^−1^) showed detectable enzymatic activities. The low rates observed, however, prevented a determination of kinetic constants. In order to investigate, whether the respective substrates bind to the enzymatic metal center in a productive manner, spectra of Hms (0.5 mM) in the presence of excessive substrate concentrations (10 mM) were recorded under anaerobic conditions. Bidentate binding of the monoanionic α-oxo acid substrate is a prerequisite for substrate decarboxylation, which is the prototypical first substrate oxidation step of α-oxo acid dioxygenases. This binding mode gives a spectroscopically accessible absorption band of low intensity in the visible region for aromatic and aliphatic α-oxo acids alike [26], [42], [43] that has previously been unambiguously assigned to a metal to ligand charge transfer transition [17]. No absorbance bands in the visible region were observed under the described conditions (data not shown), which indicates that catalytically competent complexes of enzyme with the investigated substrates did not form in substantial amounts (estimated as <10%).

**Table 1:** Specific activity of *S. coelicolor* Hms for a range of aliphatic 2-oxo acids and corresponding calculated substrate log *D* values.

*Substrate*	*Specific activity* (s^−1^)	*log D_Substrate_*
HPP	4.5	−3.6
2-Oxo-4-methylthiobutyric acid	0.007±0.001	−5.0
3-Methyl-2-oxobutyric acid	0.0006 ±0.001	−4.7
2-Oxobutyric acid	<10^−5*^	−5.0
2-Oxooctanoic acid	0.001±0.0002	−3.0
2-Oxoglutaric acid	<10^−5^	−6.2
4-Methyl-2-oxopentanoic acid	<10^−5^	−4.2
2-Oxovaleric acid	<10^−5^	−4.6

Measurements were performed in air saturated 20 mM Tris buffer at pH 7.5 and 25°C. Values for HPP are given in comparison. The limit of detection under assay conditions was a specific rate of 10^−5^ s^−1^.

The reactivity of Hms towards a range of aromatic oxo acids was characterized using a range of phenylpyruvate (PP) analogues with varying substituents in the para position of the aromatic ring. Therefore, *p*-fluoro-, *p*-methyl- and *p*-methoxy-PP were chemically synthesized (see Material and Methods section and Protocol S1 for details) and used along with commercially available compounds. In the presence of aromatic substrates Hms generally showed dioxygen consumption activities that were higher than for aliphatic substrates. Steady state enzyme kinetic analyses were consequently performed and the apparent turnover numbers (*k*_cat_) and Michaelis Menten constants (*K*_m_) at air saturation were determined. Generally, enzymatic activities towards PP analogues were between 5- and 100-fold lower than towards the native substrate of Hms, with highest enzymatic activities found for *p*-methoxy-PP (*k*_cat_  = 0.96 s^−1^) and PP (*k*_cat_  = 0.88 s^−1^) and with lowest apparent turnover numbers for substrates bearing an electron-withdrawing nitro- or fluoro-substituent in para position (*k*_cat_  = 0.035 s^−1^ and 0.077 s^−1^). Notably, 2-oxo-4-phenylbutanoate, which does not share the PP core-structure and is not hydroxylated at a benzylic carbon center, showed comparable enzymatic activities (*k*_cat_  = 0.17 s^−1^) as *p*-methyl-PP (*k*_cat_  = 0.13 s^−1^). Results are summarized in Table 2.

**Table 2:** Steady state kinetic constants of *S. coelicolor* Hms for a range of aromatic 2-oxo acids and corresponding product enantiopurity.

*Substrate*	*K_m_*	*k_cat_*	*Enantiomeric purity*
	(µM)	(s^−1^)	% *(S)*-enantiomer
HPP	220±80	4.5±0.5	n.d.
Phenylpyruvic acid	353±87	0.88±0.09	99.6±0.4
*p*-Methylphenylpyruvic acid	n.d.	0.13±0.02	>99*
*p*-Methoxyphenylpyruvic acid	160±70	0.96±0.12	55±2
*p*-Fluorophenylpyruvic acid	n.d.	0.077±0.004	88±2
2-Oxo-4-phenylbutanoic acid	35±20	0.17±0.02	>95*
*p*-Nitrophenylpyruvic acid	n.d.	0.035±0.007	n.d.

Apparent kinetic constants were determined at air saturation (20 mM Tris buffer, pH 7.5, 25°C).

* The analytical method did not allow a more precise determination.

### 3. Product spectrum of Hms

In order to gain insights into the impact of substrate structure on (i) the regioselectivity of C-C bond cleavage, (ii) the coupling of decarboxylation and hydroxylation and (iii) the stereoselectivity of hydroxylation, reaction products of α-oxo acid conversions by Hms were analyzed. Therefore, conversion mixtures where Hms (0.5 mM) had been incubated in the presence of the respective substrate (2.5 mM) for 16 hours were analyzed via HPLC-MS and in the case of PP also by GC-MS as outlined in the Materials and Methods section. Products were identified based on retention times, mass fingerprint (GC-MS) and, regarding carboxylate bearing substances, by their mono-anionic molecular masses (HPLC-MS). While the accordingly substituted (*R*)- and (*S*)-α-hydroxy mandelates are reporters for the native reaction, the putative formation of the respective non-hydroxylated acids implies an uncoupled decarboxylation. Aldehydes and acids that have the substrate’s aliphatic oxo acid chain shortened by two carbon atoms are indicative of C2–C3 cleavage of the substrate, whereby the respective acid is the product of aerobic oxidation of the initially formed aldehyde [17], [44]. The characterization of reaction products described in the following section focused on aromatic substrates, because products from the conversions of aliphatic substrates were generally present in too low concentrations for an unambiguous identification via HPLC-MS. Due to the apparent instability of HPP, which leads to substantial spontaneous cleavage to the aldehyde [44], the latter was not included in the analysis.

Generally, the predominant reaction products found after substrate conversions were α-hydroxy acids. No compounds that were prototypical of a C2–C3 cleavage reaction and no detectable products that were indicative of substrate decarboxylation were present in the reaction mixtures. This implies that <1% of the respective compounds were formed during conversion. An inventory showed that recovery rates of the reaction products as α-hydroxy acids were >95% for conversions of PP, for its *p*-methyl and *p*-methoxy substituted analogues and for 2-oxo-4-phenylbutanoic acid. However, recovered *p*-fluoromandelate accounted for only ∼30% of converted substrate. Attempts to identify putative alternative products via their prototypical masses by HPLC-MS were not fruitful and no major non-assigned elution peak was present in the elution chromatograms. For *p*-nitro-PP conversion no quantitation of formed α-hydroxy acid was feasible, because the respective standards were not available and, consequently, their presence in the reaction mixture was only qualitatively confirmed based on a prominent newly formed peak that showed the expected monoanionic product mass.

Subsequently, the enantioselectivity was assessed for all conversion products where the chirally enriched standards were available. Enantioselectivities >95% were obtained for the conversion products of PP, *p*-methyl-PP and 2-oxo-4-phenylbutanoate. An exception was the product distribution regarding the *p*-fluoro-PP conversion, which gave 12% of the (*R*)-enantiomer. The most striking impact on enantioselectivity, however, was found for the conversion of *p*-methoxyphenylpyruvate, where the products were an almost racemic mixture of 45% (*R*)- and 55% (*S*)-*p*-methoxymandelate. Given the absence of significant side product formation and the high turnover number, this demonstrates the principle competence of Hms to bring about both enantiocomplementary reactions and suggests that steric interactions have a significant impact on the respective pathway. Results are summarized in Table 2.

### 4. Structure-activity relationship analysis of oxo acid conversion by Hms

#### Quantitative structure-activity relationship (QSAR) analysis of enzyme activity

Substrate properties were correlated with catalytic properties of Hms in order to gain insights into the molecular basis of the enzyme’s substrate specificity. An obvious finding during the characterization of enzyme activity was the major difference regarding aliphatic and aromatic substrates, which were consequently analyzed separately with a focus on the readily converted aromatic compounds.

DFT calculations of the reaction mechanism have previously suggested that a primary thermodynamically unfavorable one electron reduction of molecular oxygen by the iron cofactor is followed by the nucleophilic attack of the α-oxo acid via the resulting superoxide species. According to these calculations, this step, which leads to decarboxylation, is rate determining in the chemical reaction [45], [46]. QSAR analyses of biomimetic aromatic α-oxo acid complexes have found a strong positive correlation of the Hammett parameter with the velocity of decarboxylation [12], [13]. To probe, whether a similar dependance is found for the overall reaction of Hms, log *k*_cat_ was plotted against the respective Hammett parameters σ and σ_p_^+^. No meaningful trend was obtained (Table S2, Figure S6). Also, a putative correlation of log *k*_cat_ with σ_p_^−^, which would mirror the relative stabilization of negative charge in the transition state of the rate determining step, was not found (Table S2, Figure S6). It is noteworthy that, contrary to the prototypical substrate structure of Hms, the biomimetic complexes had the aromatic ring directly fused to the α-oxo acid moiety. In order to account for this structural difference compared to the PP core-structure, the parameter σ_p_* was probed, which quantitatively describes the inductive effect of a substituent. The resulting plot of log *k*_cat_ against the σ_p_* of the respective aromatic substituents, which are linked to the pyruvate core structure, again, gave no meaningful trend (Table S2, Figure S6). To investigate, whether some correlation of log *k*_cat_ with the substrates’ propensity to donate electrons was present, as previously observed in Dke1 [14], a dependance of log *k*_cat_ from the substrates’ ε_HOMO_ values was explored (Table S2, Figure S6), but, again, no trend was observed. Another energetic barrier according to DFT calculations of the reaction mechanism is the H-abstraction step, which occurs after decarboxylation. In order to probe a putative impact of this step, which will depend on the relative stabilities of the formed radicals, on the overall reaction rate of Hms, log *k*_cat_ was plotted against σ_p_^•^ (Table S2, Figure S6), but also here the results did not indicate a correlation.

**Figure 3:**
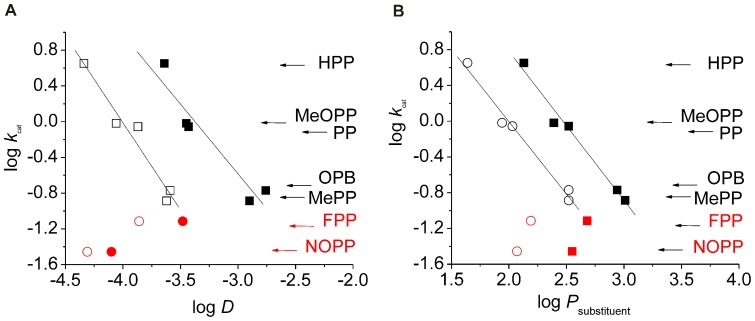
Correlation of reaction velocity with substrate and product hydrophobicity. (a.) Correlation of *S. coelicolor* Hms log *k*_cat_ values with log *D* values for the substrates phenylpyruvic acid (PP), *p*-hydroxy-PP (HPP), *p*-methoxy-PP (MeOPP), *p*-methyl-PP (MePP), 2-oxo-4-phenylbutanoic acid (OPB) (black squares). Note that *p*-fluoro-PP (FPP) and *p*-nitro-PP (NOPP, red squares) are not included in the regression analysis. The respective correlation with product log *D* is indicated by hollow circles. (b.) A correlation analogous to (a.) is depicted, whereby log *k*_cat_ is plotted against the log *P* of the respective substrate and product substituents.

Consequently, substrate properties were investigated that will not impact the chemical mechanism but may impact ligand interactions with the enzyme structure. A significant influence of substituent volume on the turnover number could easily be excluded (Table 2). Subsequently, the impact of substrate hydrophobicity was investigated. Therefore, log *D* values were calculated for all aromatic α-oxo acid substrates and plotted against log *k*_cat_. Here, a reasonable correlation of hydrophobicity with turnover number was found, except for substrates that bear electron withdrawing substituents (Figure 3 a). When log *k*_cat_ was plotted against substituent log *P*, an approach that implies that the monoanionic ligand will not change its ionization state upon the putatively rate determining interaction with the hydrophobic environment, the correlation became even clearer (Figure 3 b). While the primary substrate association velocity to the protein can in principle depend on its hydrophobicity, the found correlation with log *k*_cat_ implies that the substrate concentration is saturating and, therefore, not rate determining. Consequently, the found impact of substrate hydrophobicity on the turnover number does not mirror primary substrate association but some subsequent step. As product release is another logical step that may depend on substrate hydrophobicity, correlations were also made regarding the respective product structures and, not surprisingly, they showed similar trends (Table 2 and Figure 3). For the series of aliphatic substrates it stood out that activity was only found for substrates with a carbon chain length >6 and that log *D* values (Table 1) were, with the exception of 2-oxooctanoate, lower compared to the corresponding values of the aromatic substrates. However, no clear correlation of log *k*_cat_ with log *D* values was observed.

#### *In silico* docking analysis of enzyme substrate and enzyme product complexes

**Figure 4:**
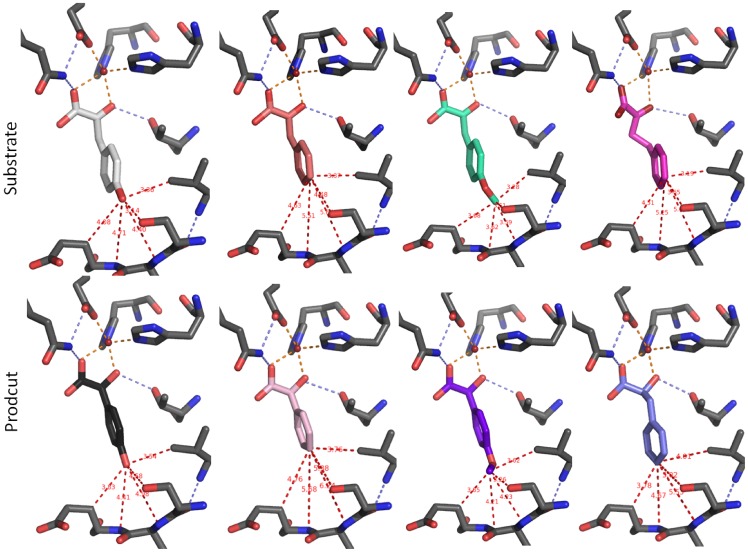
Substrate and product ligands docked into the *S. coelicolor* Hms model in silico. Several active site residues are omitted for clarity. Column 1 shows HPP (white) and (*S*)-*p*-hydroxymandelate (black), column 2 gives PP (apricot) and (*S*)-mandelate (light pink), column 3 depicts *p*-methoxy-PP (teal) and (*S*)-*p*-methoxymandelate (purple) and column 4 displays 2-oxo-4-phenylbutanoic acid (pink) and (*S*)-4-phenyllactate (light blue). Indicated distances are summarized in Table S5.

In order to gain insights into the structural basis of steric discrimination by the enzyme, in silico docking analyses were performed. Therefore, a structural model of Hms from *S. coelicolor* A3(2) was built via homology modelling based on the *A. orientalis* Hms crystal structure [10] as described in the Materials and Methods section. Although *S. coelicolor* Hms and its *A. orientalis* analogue showed the most pronounced sequence disparity among all annotated putative Hms exponents according to phylogenetic analysis [6] and sequence alignments (Figure S3), the modelled active site for the *S. coelicolor* Hms effectively overlapped with that of the *A. orientalis* Hms crystal structure and showed the same active site geometry (Figure S4). In order to test the principle applicability of the in silico docking approach, (*S*)-*p*-hydroxymandelic acid was docked into the protein structure and results were compared to the metal product complex that is present in the *A. orientalis* Hms crystal structure. The resulting ligand geometry mirrored the experimentally determined one reasonably well with an RMSD of 0.86 Å (Figure S5). Consequently, the aromatic substrate analogues were docked into the Hms model structure. The monoanionic oxo acids were generally well accommodated in the active site pocket and adopted a similar orientation with the lowest energy conformation displaying the expected bidentate coordination of the oxo and carboxylate moieties to the Fe(II) ion and with the carboxylate moiety being H-bonded (3.46 Å) to residue Gln325. Substituents had no significant impact on the orientation of the aromatic ring. Distances of the methyl and fluoro substituents were the same as those of the hydroxy group, when measured from the respective heavy atoms, C, F and O, with differences <0.1 Å. Also the methoxy substituent of *p*-methoxy-PP showed the same positioning of its oxygen atom. Its terminal carbon atom, however, protruded deeper into the active site pocket and showed decreased distances to Val223 (3.28 Å versus 3.51 Å), Ser208 (3.25 Å versus 3.51 Å), Ser221 (4.06 Å versus 3.35 Å) and Ser209 (4.44 Å versus 3.50 Å) compared to the terminal heavy atoms of HPP. Docking of (*S*)-mandelic acid and its derivatives gave analogous results, whereby the substituents of the products generally showed less protrusion into the binding pocket compared to the respective substrates. Cartoons of the lowest energy structures of substrates and products are summarized in Figure 4 and the respective distances are furthermore summarized in Table S5.

**Figure 5:**
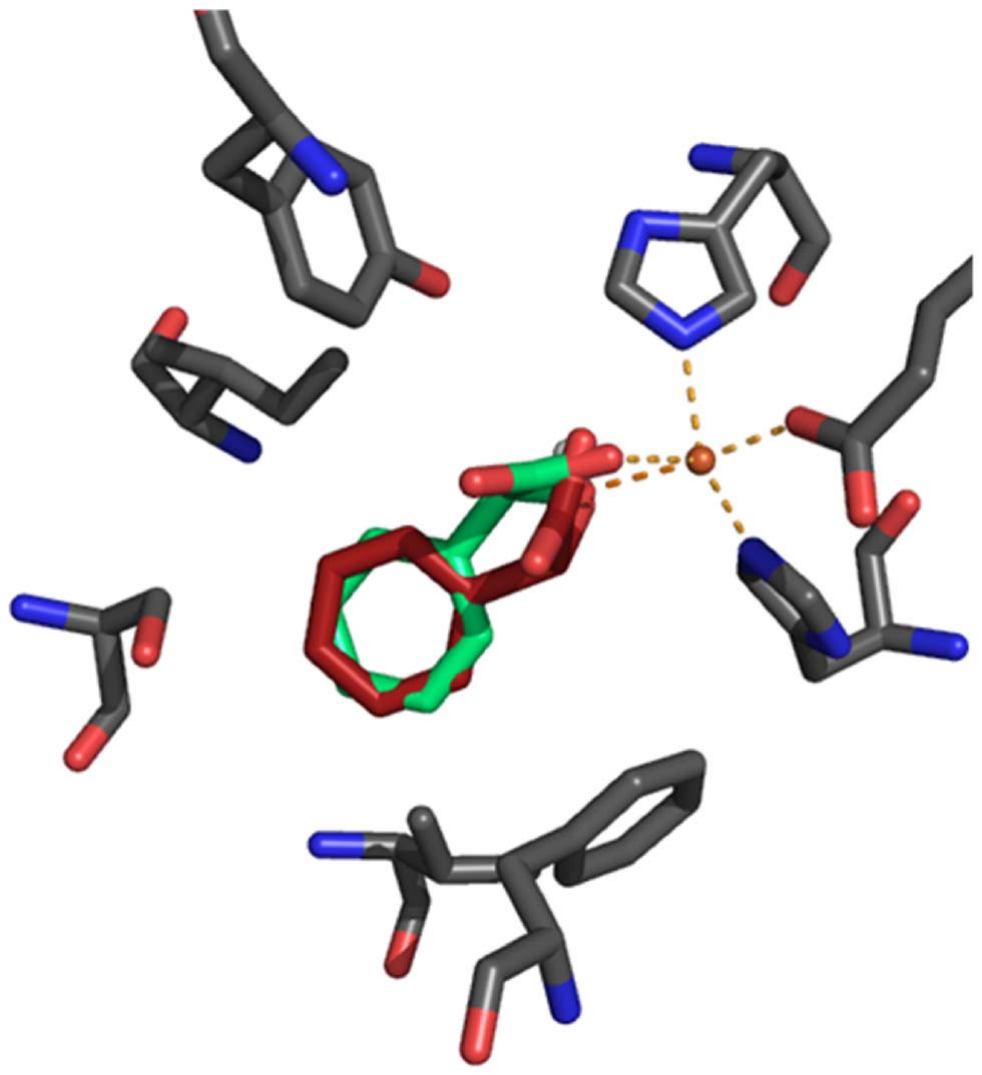
Overlay of (*R*)-mandelate and (*S*)-mandelate ligands docked into the *S. coelicolor* Hms model in silico. The (*S*)-mandelate (green) coordinated structure showed a slightly distorted trigonal bipyramidal geometry that mirrors crystal structural data of the *A. orientalis* HMS product complex (PDB: 2R5V) with angle deviations <2°. In the (*R*)-mandelate (red) bound model, the positions of the axial ligands, namely the carboxylate oxygen from Glu340 and the hydroxy oxygen from mandelate, were preserved. However, the residues that form the trigonal plane showed major deviations from ‘ideal’ geometry: Angles between the coordinated (*R*)-mandelate’s carboxylate oxygen, the iron cofactor and the histidines’ metal-nitrogens shifted from 110° to 139° (His 181) and from 124° to 100° (His261), respectively.

It is noteworthy that the crystal structure of *A. orientalis* Hms shows the product ligand in a slightly distorted trigonal bipyramidal coordination, whereby the hydroxy moiety at C-2 occupies an axial position. According to DFT calculations a trigonal bipyramidal iron center is one of two feasible geometries that the hydrogen abstracting Fe(IV) = O species can adopt, and the subsequent rebound reaction then proceeds in the respective geometry [47]. This implies that the trigonal bipyramidal product bound metal center may approximate the transition state of the ultimate step of hydroxylation, which determines the product’s chirality. Consequently, in order to probe to what extent the active site of Hms may discriminate the binding of the two product enantiomers, the respective (*R*)-hydroxy acids were also docked into the active site. The ligands adopted an overall binding geometry that resembled that of their enantiomeric counterparts and the positions of the aromatic rings entirely overlapped, as demonstrated in the structural overlay of (*R*)-and (*S*)-mandelate (Figure 5). Interestingly, upon closer inspection we found that the (*S*)-product ligated structures generally showed a geometry that mirrored trigonal bipyramidal geometries with angle deviations <2° from the crystal structure metal center, while binding geometries of the respective (*R*)-mandelate complexes were siginifcantly distorted (Figure 5). Calculated binding energies for all docked ligands are given in the Supporting Information (Table S3 and Table S4). However, we caution that the calculated binding energies can at best be a very rough estimate of relative affinities.

## Discussion

### The impact of substrate structure on enzymatic activity

Previous DFT calculations of the Hms reaction coordinate suggest that the rate determining step of the chemical mechanism is the nucleophilic attack of the substrate’s keto-group by a formed superoxide species [45]. In contrast to biomimetic complexes [12], [13], however, no corresponding correlation was obtained for *S. coelicolor* Hms. Also, a correlation which would indicate that the preceding step of oxygen activation is rate determining was not found. In principle, the impact of substituents on both steps could be weaker than in the respective model systems due to the distinct substrate structures, or, alternatively, both effects might compensate one another in the enzymatic reaction. However, in such a scenario similar turnover numbers for the tested substrates would be expected, which is not observed. Instead, a pronounced correlation of log *D* with log *k*_cat_ is found. The latter implies that a noncatalytic step in the cycle determines the overall reaction rate for the substrates that fall into this correlation, with the upper and lower limits of *p*-hydroxy-PP and *p*-methoxy-PP, respectively. *k*_cat_ by definition does not mirror the primary substrate association event. An alternative, secondary binding event that is rate determining can, however, not be excluded with certainty based on our results. Kinetically discernible ligand coordination at a mononuclear iron center subsequent to substrate association has previously been reported and its rate was independent of the substrate concentration. However, this step was governed by electronic effects [28]. We, therefore, consider product release to be the logical rate-limiting step of the overall conversion regarding the substrates that are part of the log *k*_cat_ – log *P* correlation, while for the substrates with electron withdrawing substituents, namely *p*-fluoro-PP and *p*-nitro-PP, obviously some other step becomes rate limiting. Structural analysis of the *S. coelicolor* Hms model (see Materials and Methods section for details) reveals one major channel that travels through the protein and incorporates the active site (Figure S7). One side of the tunnel structurally corresponds to the comparably wide opening found in plant Hppds [48], and it consists of an ‘inner’ hydrophobic part (Ile238, Ile247, Phe327, Phe338, Leu 358, Tyr 359 and Val362) and an ‘outer’ hydrophilic section (Glu365, Arg366, Gln349). The other side comprises the substrate binding pocket, which is connected with the bulk via a small gate (Ser208, Ser209, Gu210, Ser221, Val223, Ile236, Phe350, Gly351, Ser352 and Ile355). The described tunnel, which is also present in the *A. orientalis* structural model, constitutes the logical route of substrate- and product trafficking in Hms and its hydrophobic features may indeed make it ‘sticky’ for hydrophobic ligands.

### The impact of substrate and protein structure on the enantioselectivity of hydroxylation

Hms from *S. coelicolor* A3(2) oxidizes PP to (*S*)-mandelate with high enantiomeric purity ( 99.6%). This is in line with findings regarding *A. orientalis* Hms, for which high enantioselectivity was reported, although no actual value was given [3]. In the current study, a high (*S*)-selectivity was found for most investigated substrates. A remarkable exception was the oxidation of the *p*-methoxy substituted PP analogue, which gave an almost racemic mixture of products. This suggests that in this case the reaction intermediate during the hydroxylation step of catalysis is distinctly oriented. Notably, recovery of the respective products as *p*-methoxymandelate was >95%, which implies that the decarboxylation and hydroxylation step remain strongly coupled, despite the probable displacement of the intermediate from the native binding geometry. Enantioselectivity of *p*-fluoro-PP conversion is also decreased, which indicates some interference of the fluoro substituent with intermediate positioning, possibly by its potential to form H-bonds. Products of oxygenative C2-C3 cleavage or decarboxylation were not observed. Due to the incomplete recovery of products, however, the formation of intermediates that may result from ring oxygenation cannot be ruled out for *p*-fluoro-PP conversion.

**Figure 6:**
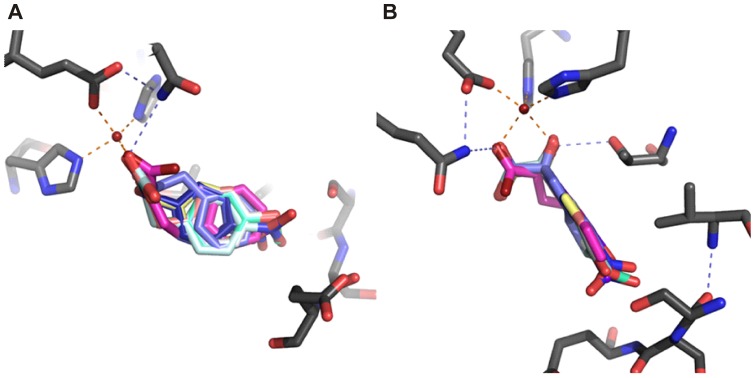
Overlay of aromatic substrates and products docked into the *S. coelicolor* Hms model in silico. The lowest energy conformations of aromatic α-oxo substrates and corresponding (*S*)-hydroxy products are shown. Several active site residues are omitted for clarity. Panel (a.) shows a perspective normal to the plane that the aromatic rings occupy; panel (b) gives a perspective parallel to the plane.

An analysis via in silico docking suggests that the aromatic substrates as well as the respective products all adopt a similar orientation in the active site (Figure 4). In the case of the *p*-methoxy substituted ligands the substituent protrudes further into the active site pocket, an interaction that may impact intermediate positioning during catalysis. An overlay of aromatic products and substrates that had been docked into the active site of *S. coelicolor* Hms shows that the aromatic ring occupies a well defined space in the structure, as demonstrated in Figure 6. Notably, aliphatic substrates were not well converted. The energetically unfavorable H-abstraction of nonbenzylic hydrogens [49] did not play a significant role regarding the low conversion rates, given that 2-oxo-4-phenylbutanoic acid, which presents both, a non-activated carbon atom and an aromatic ring, is efficiently converted. Binding studies, furthermore, showed that the aliphatic compounds did not form spectroscopically accessible catalytically competent primary complexes.

Based on these observations we propose that the hydrophobic ligand binding pocket stabilizes aromatic substrates compared to their aliphatic counterparts and is responsible for the enzyme’s high selectivity towards aromatic substrates. The active site of Hms readily accommodates the (*S*)-enantiomeric products in this pocket whereby it allows them to coordinate to the metal center in a trigonal bipyramidal binding mode. This geometry is presumably present during the transition state of hydroxylation and, interestingly, it is compromised when the enantiocomplementary ligands are docked into the hydrophobic pocket. Whether this observation has actual significance for the enantioselectivity of catalysis in Hms is the focus of ongoing studies.

Its catalytic properties make Hms an interesting, potential tool for the biocatalytic production of a range of aromatic chiral α-hydroxy acids. In silico docking analyses suggest that the size of the substrate binding pocket may set a limit to the enzyme’s (*S*)-enantioselectivity with respect to the conversion of bigger substrates. An in depth understanding of the molecular basis of regio- and stereoselective catalysis at mononuclear nonheme iron centers will be key to establishing them as a platform for tailor made, O_2_ dependent oxidations, a reaction type with high synthetic potential [50].

## Supporting Information

Figure S1 **SDS gel electrophoretic analysis of purified Hms preparations.** Lane 1 shows the molecular weight standard, lanes 2 and 3 show purified C-terminally tagged Hms in the presence (lane 2) and absence (lane 3) of DTT, while in lanes 4 and 5 the respective N-terminally tagged Hms preparations in the presence and absence of DTT are displayed.
(PDF)

Figure S2 **Gel filtration chromatogram of purified Hms.** The elution trace (280 nm) of C-terminally tagged Hms preparations shows a homogenous species with an estimated mass of ∼45 kDa. The dashed line indicates the elution profile of the molecular weight standard with the proteins thyroglobulin (670 kDa), γ-globulin (158 kDa), ovalbumin (44 kDa), myoglobin (17 kDa), and vitamin B12 (1.355 kDa) from left to right.
(PDF)

Figure S3 **Alignment of putative Hms sequences.** Based on conserved sequence motifs 24 proteins with putative Hms activity were identified from the NCBI data base. Metal binding sites are in cyan, conserved active site residues are in pink (strong conservation) and deviations from the conserved motif are indicated in yellow. The entry of the *S. coelicolor A3(2)* sequence is also marked in yellow.
(PDF)

Figure S4 **Superposition of** ***A. orientalis*** **Hms crystal structure (teal) and** ***S. coelicolor*** **Hms model (grey).** The metal ion is in orange, the metal bound (S)-*p*-hydroxymandelate ligand, which is present in the crystal structure, is shown in pink. Note that the substitution of phenylalanine (Phe188, *A. orientalis*) by serine (Ser208, *S. coelicolor* A3(2)), which is the only noticeable difference between both active sites, is indicated with a red circle and does not significantly change the geometry of the substrate binding cavity.
(PDF)

Figure S5 **Validation of the in silico docking method.** Overlay of the product (S)-*p*-hydroxymandelate complex from the *A. orientalis* crystal structure and the corresponding product *S. coelicolor* Hms model complex from in silico docking (orange). Ligands coincide with an RMSD value of 0.86 Å.
(PDF)

Figure S6 **Correlations of log** ***k*** **_cat_ with QSAR parameters from Table S2.** Plots of (A.) log *k*_cat_ vs. σ (B.) log *k*_cat_ vs. σp• (C.) log *k*_cat_ vs. σp+ (D.) log *k*_cat_ vs. σp− (E.) log *k*_cat_ vs. σ* (F.) log *k*_cat_ vs. εHOMO.
(PDF)

Figure S7 **Model of** ***S. coelicolor*** **A3(2) Hms with its putative substrate/product trafficking tunnel.** With Tyr359 positioned in an alternative low energy conformation, a channel opens up that travels through the enzyme without a barrier. (A.) Perspective of the long part of the tunnel that leads from the active site to the bulk along the N-terminal α-helix and is lined by Tyr359. (B.) Perspective of the short part of the tunnel that leads from the active center outside via a gate lined by Ser 208. (C.) Perspective from (A.) with the product ligand modeled into the active site. (D.) Perspective from (B.) with product ligand bound. (E.) Structural model with the channel indicated by a red arrow. (F.) Structural model from (E.) in the presence of ligand and with the molecular protein surface hidden. The iron cofactor is shown as a pink sphere. The protein fold is shown as a ribbon (β-sheets in red, α-helices in blue). Channel gating residues Ser208 and Tyr359 are shown. The molecular surface of the protein is displayed in transparent green. Note that in the *A. orientalis* Hms model an analogous channel is present (not shown), whereby Tyr339 and Phe188, which correspond to Tyr359 and Ser208 in *S. coelicolor* Hms, adopt low energy conformations that open and close the tunnel entrances.
(PDF)

Table S1 **Biochemical characteristics of C- and N-terminally tagged** ***S. coelicolor*** **Hms.** Specific activities were determined with HPP (5 mM) as a substrate in air saturated buffer (Tris 20 mM, pH 7.5, 25°C). Parameters were determined as outlined in the Material and Methods section.
(DOCX)

Table S2 **Parameters employed for QSAR analysis.** σ is the sigma parameter as defined by Hammett; σ_p_^•^, σ_p_^+^, σ_p_^−^ are sigma para values derived from models that have radical character or positive or negative charge during the respective reaction’s transition state; σ^*^ quantitatively describes aliphatic inductive effects [33]. The frontier energy of each monoanionic ligand’s highest occupied molecular orbital ε_HOMO_ and the atomic volume (MV) of the substituent that is fused to the α-keto-pyruvate core structure were both obtained from DFT calculations using the software Spartan (Wavefunction Inc.). * n.a. indicates that no parameter was available.
(DOCX)

Table S3 **Theoretical binding energies of monoanionic aliphatic substrates from in silico docking experiments.**
(DOCX)

Table S4 **Theoretical substrate and product binding energies of monoanionic aromatic ligands from in silico docking experiments.**
(DOCX)

Table S5 **Distances of aromatic ligand substituents to residues in the** ***S. coelicolor*** **Hms active site pocket derived from in silico docking studies.** Distances (d) to the respective closest heavy atoms are given, as indicated in Figure 4.
(DOCX)

Protocol S1 (DOC)

Protocol S2 (PDF)
