# Supplementary figures and images for: Chiral Hydroxylation at the Mononuclear Nonheme Fe(II) Center of 4-(S) Hydroxymandelate Synthase – A Structure-Activity Relationship Analysis
Source: PLoS One. 2013 Jul 23;8(7):e68932. doi: 10.1371/journal.pone.0068932 (PMC3720870; doi:10.1371/journal.pone.0068932)

MM

1

2

3

4

5

[kDa]

97.0

66.0

45.0

30.0

20.1

14.4

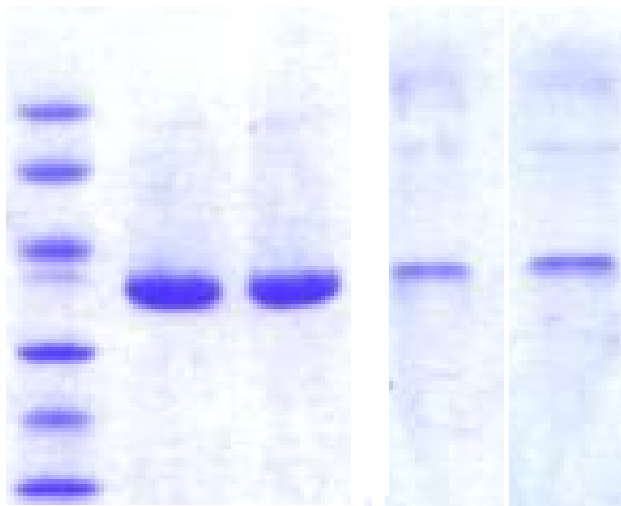

Supplement: Figure S1 — SDS gel electrophoretic analysis of purified Hms preparations. Lane 1 shows the molecular weight standard, lanes 2 and 3 show purified C-terminally tagged Hms in the presence (lane 2) and absence (lane 3) of DTT, while in lanes 4 and 5 the respective N-terminally tagged Hms preparations in the presence and absence of DTT are displayed. (PDF) [file pone.0068932.s001.pdf]

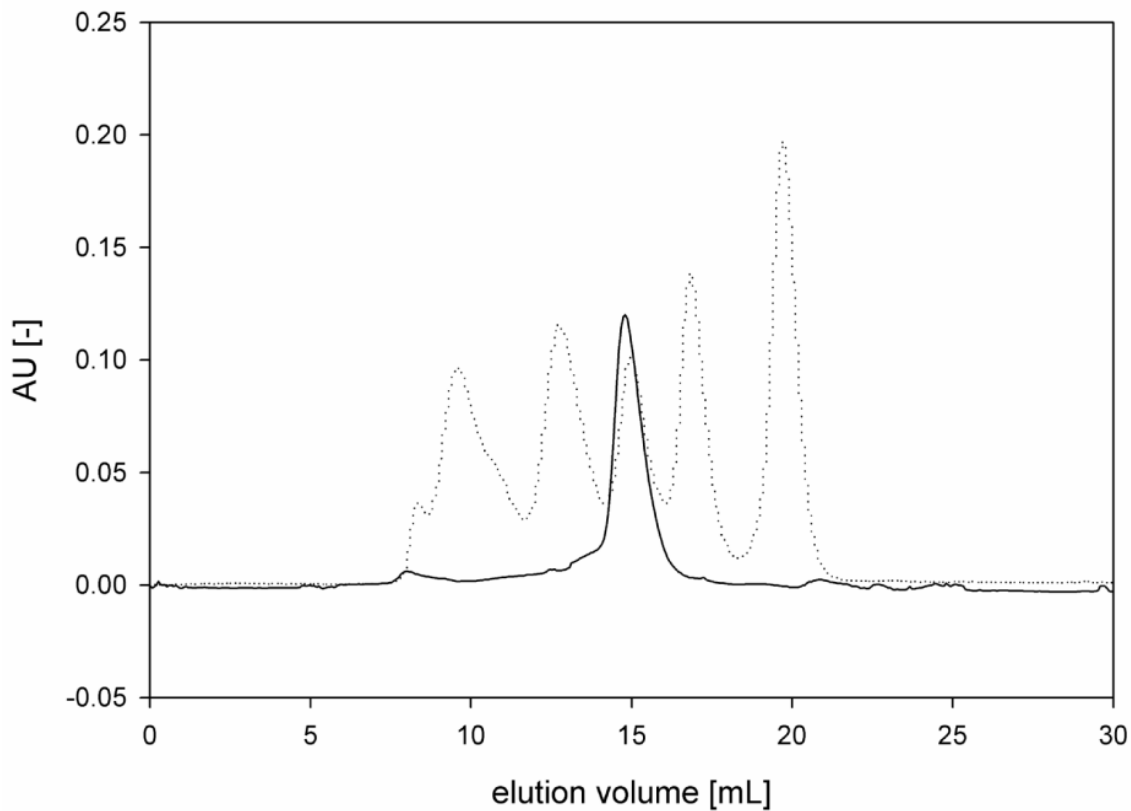

Supplement: Figure S2 — Gel filtration chromatogram of purified Hms. The elution trace (280 nm) of C-terminally tagged Hms preparations shows a homogenous species with an estimated mass of ∼45 kDa. The dashed line indicates the elution profile of the molecular weight standard with the proteins thyroglobulin (670 kDa), γ-globulin (158 kDa), ovalbumin (44 kDa), myoglobin (17 kDa), and vitamin B12 (1.355 kDa) from left to right. (PDF) [file pone.0068932.s002.pdf]

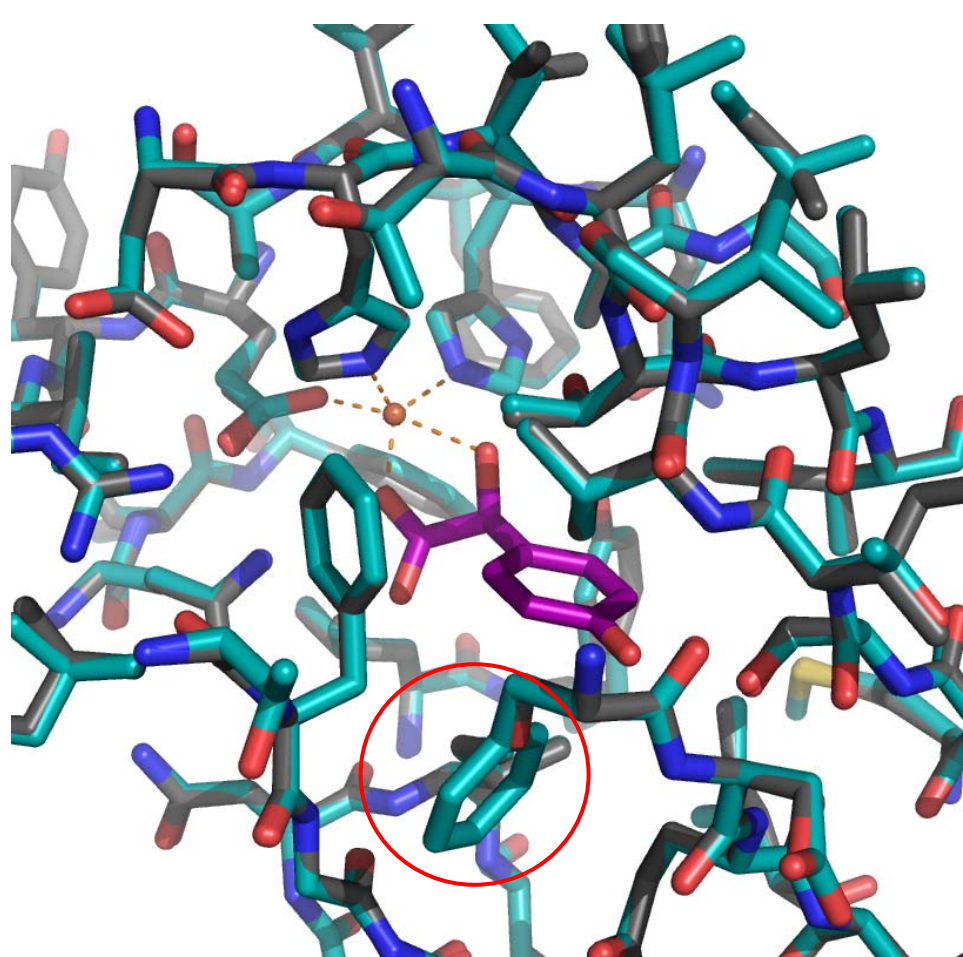

Supplement: Figure S4 — Superposition of A. orientalis Hms crystal structure (teal) and S. coelicolor Hms model (grey). The metal ion is in orange, the metal bound (S)-p-hydroxymandelate ligand, which is present in the crystal structure, is shown in pink. Note that the substitution of phenylalanine (Phe188, A. orientalis) by serine (Ser208, S. coelicolor A3(2)), which is the only noticeable difference between both active sites, is indicated with a red circle and does not significantly change the geometry of the substrate binding cavity. (PDF) [file pone.0068932.s004.pdf]

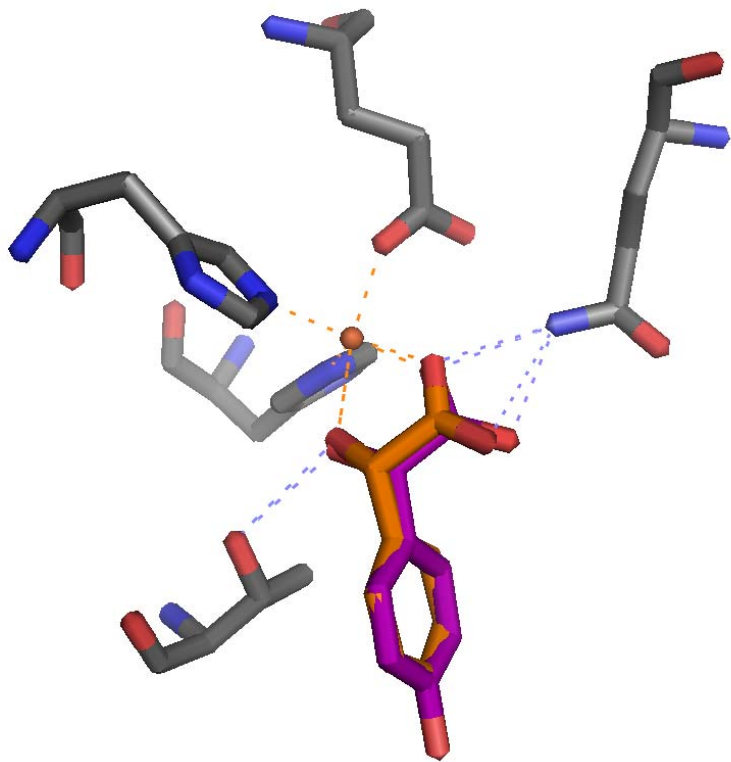

Supplement: Figure S5 — Validation of the in silico docking method. Overlay of the product (S)-p-hydroxymandelate complex from the A. orientalis crystal structure and the corresponding product S. coelicolor Hms model complex from in silico docking (orange). Ligands coincide with an RMSD value of 0.86 Å. (PDF) [file pone.0068932.s005.pdf]

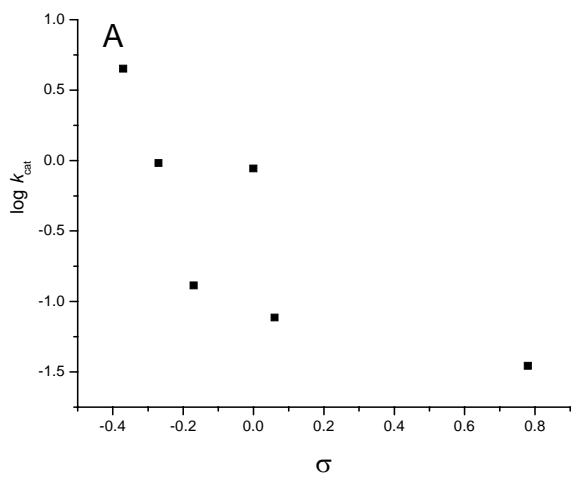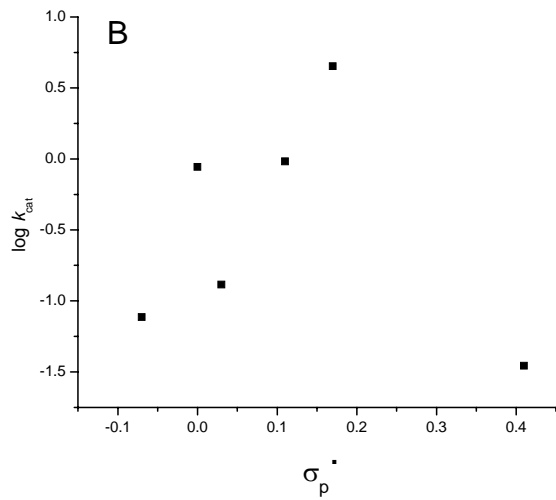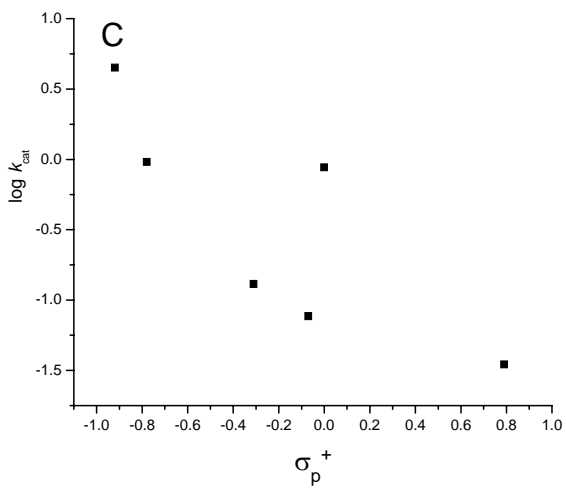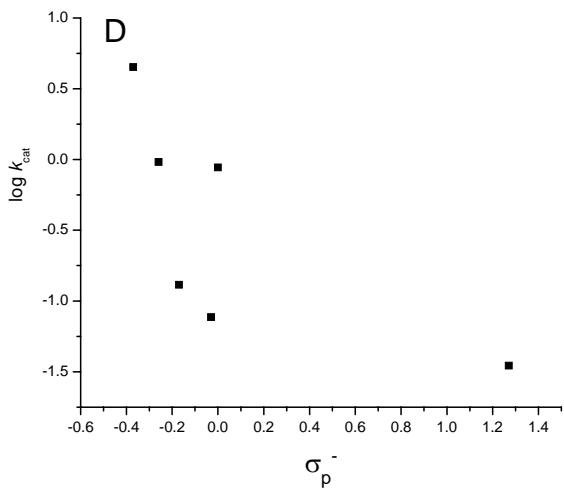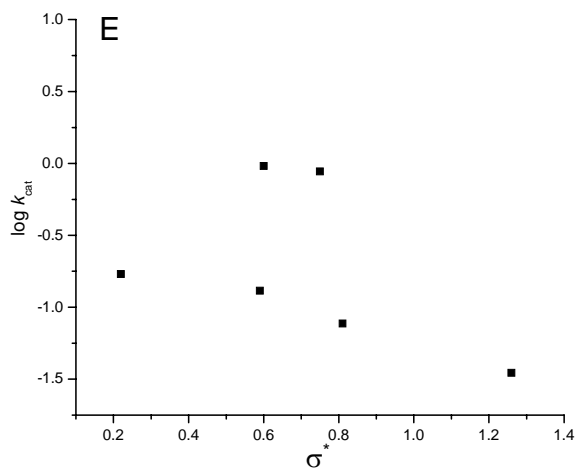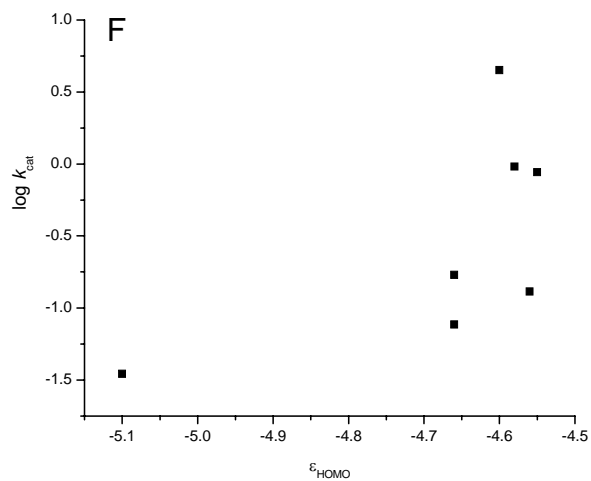

Supplement: Figure S6 — Correlations of log k cat with QSAR parameters from Table S2. Plots of (A.) log k cat vs. σ (B.) log k cat vs. σp• (C.) log k cat vs. σp+ (D.) log k cat vs. σp− (E.) log k cat vs. σ* (F.) log k cat vs. εHOMO. (PDF) [file pone.0068932.s006.pdf]

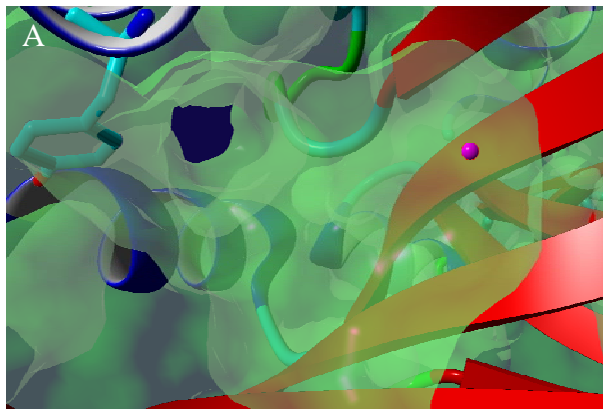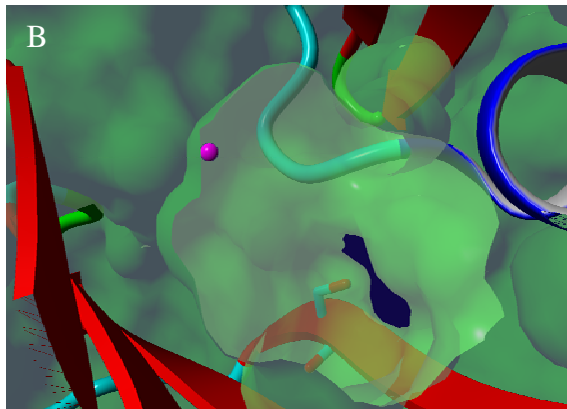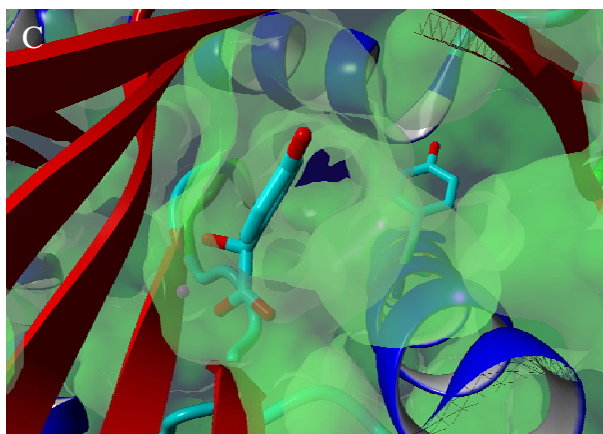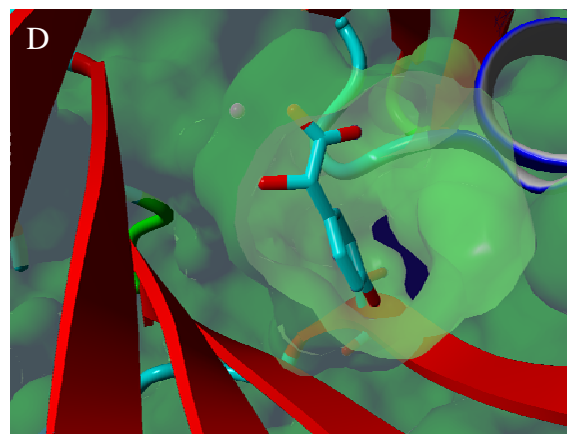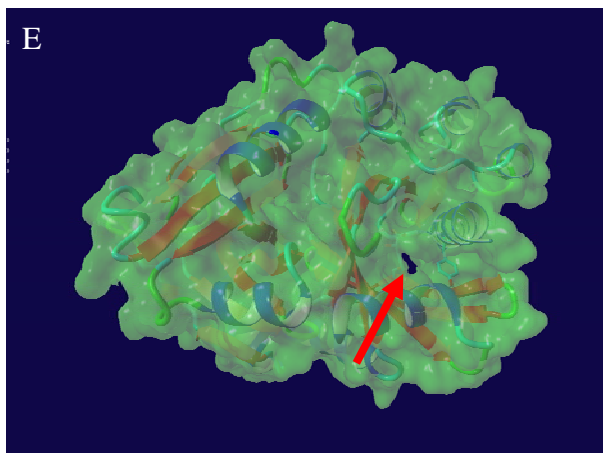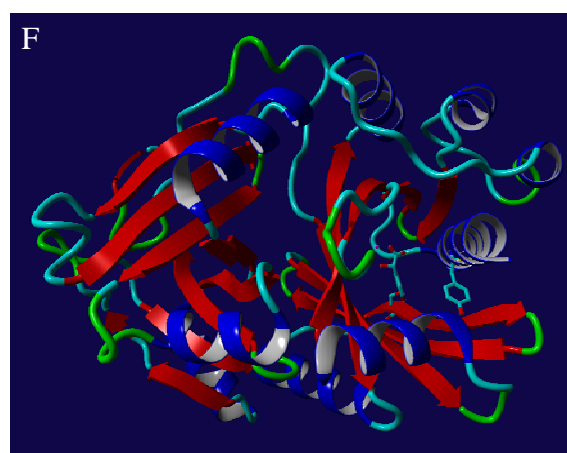

Supplement: Figure S7 — Model of S. coelicolor A3(2) Hms with its putative substrate/product trafficking tunnel. With Tyr359 positioned in an alternative low energy conformation, a channel opens up that travels through the enzyme without a barrier. (A.) Perspective of the long part of the tunnel that leads from the active site to the bulk along the N-terminal α-helix and is lined by Tyr359. (B.) Perspective of the short part of the tunnel that leads from the active center outside via a gate lined by Ser 208. (C.) Perspective from (A.) with the product ligand modeled into the active site. (D.) Perspective from (B.) with product ligand bound. (E.) Structural model with the channel indicated by a red arrow. (F.) Structural model from (E.) in the presence of ligand and with the molecular protein surface hidden. The iron cofactor is shown as a pink sphere. The protein fold is shown as a ribbon (β-sheets in red, α-helices in blue). Channel gating residues Ser208 and Tyr359 are shown. The molecular surface of the protein is displayed in transparent green. Note that in the A. orientalis Hms model an analogous channel is present (not shown), whereby Tyr339 and Phe188, which correspond to Tyr359 and Ser208 in S. coelicolor Hms, adopt low energy conformations that open and close the tunnel entrances. (PDF) [file pone.0068932.s007.pdf]
