# Supplementary material for: Chiral Hydroxylation at the Mononuclear Nonheme Fe(II) Center of 4-(S) Hydroxymandelate Synthase – A Structure-Activity Relationship Analysis
Source: PLoS One. 2013 Jul 23;8(7):e68932. doi: 10.1371/journal.pone.0068932 (PMC3720870; doi:10.1371/journal.pone.0068932)
Supplement: Figure S3 — Alignment of putative Hms sequences. Based on conserved sequence motifs 24 proteins with putative Hms activity were identified from the NCBI data base. Metal binding sites are in cyan, conserved active site residues are in pink (strong conservation) and deviations from the conserved motif are indicated in yellow. The entry of the S. coelicolor A3(2) sequence is also marked in yellow. (PDF) [file pone.0068932.s003.pdf]

56248

Sequence	Start	Alignment	End
2R5V_AChain A, Hydroxymandelate Synthase Crystal Structure	128	QR-DGTSAE--LPPGFTGSMD----VTNHGKGDVDLLGIDHFAICLNAGDLGPTVEYYERALGFRQIFD---EHIVVGAQ	197
ACJ60972VEG30 [uncultured soil bacterium]	128	QR-DGTSAE--LPPGFTGSMD----VTNHGKGDVDLLGIDHFAICLNAGDLGPTVEYYERALGFRQIFD---EHIVVGAQ	197
CAC48371putative hydroxyphenyl pyruvate dioxygenase [Amycolatopsis balhimycina]	128	QR-DGSAPQ--LPPGFTGSLE----VTNHGRGDVDLLGIDHFAICLTAGDLVPTVEYYERALGFRQIFD---EHIVVGSQ	197
AAM80551HmaS [Streptomyces toyocaensis]	134	QS-GEATAA--AP-------E----TTGQGGDVNLLGLDHFAVCLNSGDLGPTVAFYERAFGFRQIFE---EHIVVGRQ	196
CAD91197putative hydroxymandelate synthase [Nonomuraea sp. ATCC 39727]	143	QRAPGEEPG--LPAGFS-PIS----RTGDGPAGVDLLDLDHIAVCLNTGDLDPTVDHYLTAFGFRRIFE---ERIVVGRQ	212
AAK81835hydroxyphenyl pyruvate dioxygenase [Streptomyces lavendulae]	132	QRDPGDDPG--LPVGFSEAPS----AAESGADAAELLDIDHFAVCLPTGDLDIITDFYVATLGFSETFK---ERIEVGTQ	202
ZP_046968484-hydroxyphenylpyruvate dioxygenase [Streptomyces roseosporus NRRL 15998]	112	EREPGASGG--LPAGFTPVAN----EEPRWAGEVGLLDIDHFAVCLPAGDLETTVRFYRNALGFSEIFE---ERIVVGAQ	182
YP_0033424144-hydroxyphenylpyruvate dioxygenase [Streptosporangium roseum DSM 43021]	135	QRREGVPAD--RLPGFV-PAS----GREAAAPA-GLLEIDHFAVCLEPGELRSVVEFYERVLDFRNTFT---EKIVVGAQ	203
CAG15040HmaS protein [Actinoplanes teichomyceticus]	138	Q-RAGDTDE-RALPGLRPVYG------SASGTGGNLDEVDHFAVCVESGQIDATVDFYRHILDFELIFT---EHIVVGSQ	206
CAE53378HmaS protein [Actinoplanes teichomyceticus]	132	QREPGGPWS---LPGLN-PV------HRPGTPGIGLRLVDHFAVCVEAGRLTEVVEHYERVFGFSAIFT---ERIVVGEQ	198
YP_0031022694-hydroxyphenylpyruvate dioxygenase [Actinosynnema mirum DSM 43827]	130	QREPGGPWS---LPGLN-PV------HRPGTPGIGLRLVDHFAVCVEAGRLTEVVEHYERVFGFSAIFT---ERIVVGEQ	196
YP_0011595494-hydroxyphenylpyruvate dioxygenase [Salinispora tropica CNB-440]	134	QRPGGAP------------------GEDPESAG-GLRVLDHFAVCLEAGGLEPTVAFYQEVLDFRVVFE---EKIVVGAQ	191
YP_0033838684-hydroxyphenylpyruvate dioxygenase [Kribbella flavida DSM 17836]	138	QGRAG-------MIGVVPAEGPG-NRRSTSGADGELGEIDHFAVCVYAGDLDTTVSFYRDVLDFELIFA---ERVQVGSQ	206
ZP_064002134-hydroxyphenylpyruvate dioxygenase [Micromonospora sp. L5]	135	ER-HGPRRE--FLPGAFQITEP-----AGGPDEKLFSEIDHLAICVPSGQLKPTVDFYENVFGFLPIFD---EYIEVAGQ	203
ZP_062154194-hydroxyphenylpyruvate dioxygenase [Micromonospora aurantiaca ATCC 27029]	134	ER-RGDPGV--FLPGAIEPVTA-----PDGTDT-LLAEVDHLAICVPPGQLDETVAHYEKVFGFADIFT---EHIEVAGQ	201
YP_4815504-hydroxyphenylpyruvate dioxygenase [Frankia sp. CcI3]	134	ER-RGDPGV--FLPGAIEPVTA-----PDGTDT-LLAEVDHLAICVPPGQLDETVAHYEKVFGFADIFT---EHIEVAGQ	201
ZP_055255754-hydroxyphenylpyruvic acid dioxygenase [Streptomyces lividans TK24]	134	SR-EAVEGP--FSPGLIE-ETV-----PDRSNEGLLRAIDHVAVCLPAGELHPTVRAYRDVFGFTRTFE---ERIVVGSQ	201
NP_6274424-hydroxyphenylpyruvic acid dioxygenase [Streptomyces coelicolor A3(2)]	134	PAADGDGAG--LLPPDRDWALLP----AATGRTGPRPLLDHVAVCLESGTLRSTAEFYEAAFDMPYYSS---EYIEVGEQ	204
YP_0031140044-hydroxyphenylpyruvate dioxygenase [Catenulispora acidiphila DSM 44928]	147	PAADGDGAG--LLPPDRDWALLP----AATGRTGPRPLLDHVAVCLESGTLRSTAEFYEAAFDMPYYSS---EYIEVGEQ	217
ABD659454-hydroxyphenylpyruvate dioxygenase [Streptomyces fungicidicus]	134	ER---RGAA--FWPGAIEPQPAP-----ARTEPELVRAIDHAAVLVPDGELEPTVEYYQRVFGFKLIFE---EYVEVGAQ	200
YP_0011593144-hydroxyphenylpyruvate dioxygenase [Salinispora tropica CNB-440]	134	SR-EDPGAP--FAP-FIE-ETG-----AHGSG-GMLKRVDHFAVCVPAGELDGTVRRYQEVFGLSQTFE---ERIVVGSQ	199
YP_0015375014-hydroxyphenylpyruvate dioxygenase [Salinispora arenicola CNS-205]	156	ER-RRGGRD--FLPGLAELPPV-----AETAED-LLVEIDHLAVCVPPGQLAETVCGYREVFGFDEIFH---EYVEVGGQ	223
ZP_046041114-hydroxyphenylpyruvate dioxygenase [Micromonospora sp. ATCC 39149]	134	ER-RRGGPD--FLPGLAELPPV-----DDTAEN-LLAEIDHLAVCVPPGQLAETVRGYREVFGFAEIFH---EYVEVDGQ	201
	31	QR-RGDRRD--FLPGAFESAEP-----AGGADG-LLAEIDHLAICVPPGQLDATVAHYERVFGFAQIFT---EHIEVAGQ	98

*-x

x- x +

56248

Sequence	Start	Alignment	End
2R5V_AChain A, Hydroxymandelate Synthase Crystal Structure	198	AMNSTVVQSASGAVTLTLIEPDRNADPGQIDEFLKDHQGAGVQHIAFNSNDAVRAVKALSERGVEFLKTPGAYYDL--LG	275
ACJ60972VEG30 [uncultured soil bacterium]	198	AMNSTVVQSASGAVTLTLIEPDRNADPGQIDEFLKDHQGAGVQHIAFNSNDAVRAVKALSERGVEFLKTPGAYYDL--LG	275
CAC48371putative hydroxyphenyl pyruvate dioxygenase [Amycolatopsis balhimycina]	198	AMNSTVVQSVSGAVTLTLIEPDRNADPGQIDEFLKGHQGAGVQHIAFNSDDAVRAVKALSERGVAFLKTPGAYYDM--LG	275
AAM80551HmaS [Streptomyces toyocaensis]	197	AMNSTVVQSASGEVTLTLIEPDSNADPGQIDEFLKAHQGAGVQHIAFNADDAVRAVRALSGRGVEFLKTPGTYYDM--LG	274
CAD91197putative hydroxymandelate synthase [Nonomuraea sp. ATCC 39727]	213	AMESAVVQSPSGSVTLTLIQPDPSADPGQIDEFLKAHQGAGVQHLAFSSPDAVRSVRALADRGVTFLTTPQAYYDL--LG	290
AAK81835hydroxyphenyl pyruvate dioxygenase [Streptomyces lavendulae]	203	AMESKVVQSASGAVTLTLIEPDPMAEAGQIDMFLERHAGAGVQHVAFSSSDAVHAVNTLSERGVRFLSTPGSYYDL--LE	280
ZP_046968484-hydroxyphenylpyruvate dioxygenase [Streptomyces roseosporus NRRL 15998]	183	AMESKVVQSPNGVVTLTLIEPDTKAEPGQIDDFLKNHDGAGVQHIAFSSDDAVRSVRALRERDVRFLKTPDAYYDL--LD	260
YP_0033424144-hydroxyphenylpyruvate dioxygenase [Streptosporangium roseum DSM 43021]	204	AMDSEVVQNPSGTVTLTLIEPDTRREPGQIDFFIKNHGGAGVQHIAFRTEDIVASVGTMRSRGVDFLSTPATYYHM--LG	281
CAG15040HmaS protein [Actinoplanes teichomyceticus]	207	AMTIKVVQSRSGAVTLTLIEPDVSQVAGHIDEFLKHHGGAGVQHMAFTAGDIVEAVGTIGARGVEFLSTPDAYYSL--LP	284
CAE53378HmaS protein [Actinoplanes teichomyceticus]	199	AMDSQVVQSAGGAVTLTVIAPDTTRRPGQIDTFLKDHGGPGVQHIAFETDDVTRSVGAMSDAGIEFLTTPAAYYER--LR	276
YP_0031022694-hydroxyphenylpyruvate dioxygenase [Actinosynnema mirum DSM 43827]	197	AMDSQVVQSAGGAVTLTVIAPDTTRRPGQIDTFLKDHGGPGVQHIAFETDDVTRSVGAMSDAGIEFLTTPAAYYER--LR	274
YP_0011595494-hydroxyphenylpyruvate dioxygenase [Salinispora tropica CNB-440]	192	AMNSKVVQSTSGAVTLTLIEPDTSRKPGQIDDFIKNHGGAGVQHIAFATDGIVDAVRRLRERGVELLTTPAAYYDL--LA	269
YP_0033838684-hydroxyphenylpyruvate dioxygenase [Kribbella flavida DSM 17836]	207	AMTTKVVQSRSGTLTLTLIEPDLNCEAGHINDFLTNHRGPGVQHIAFTAECIVQAVDVIGARGVELLSTPDAYYAA--LP	284
ZP_064002134-hydroxyphenylpyruvate dioxygenase [Micromonospora sp. L5]	204	GMESTVVQSPSKHVTFTLIEPDPNRRPGQIDDFLSWHAGAGVQHIALRTSDIVEAVGTLAGRGVNFLGTPAAYYQA--LP	281
ZP_062154194-hydroxyphenylpyruvate dioxygenase [Micromonospora aurantiaca ATCC 27029]	202	AMNSKVVQSPSGTVTFVLLEPDTGARPGQIEDFLRWHAGAGVQHLGLRTDDIVTAVGALAGRGVRFASTPGAYYDG--LE	279
YP_4815504-hydroxyphenylpyruvate dioxygenase [Frankia sp. CcI3]	202	AMNSKVVQSPSGTVTFVLLEPDTGARPGQIEDFLRWHAGAGVQHLGLRTDDIVTAVGALAGRGVRFASTPGAYYDG--LE	279
ZP_055255754-hydroxyphenylpyruvic acid dioxygenase [Streptomyces lividans TK24]	202	AMDSQVVRSPSGKVTFTIIEPDTTRAPGQIDEFVRSHGGAGIQHIAFRTDDITAAVRDSAKRGVRFLTTPASYYEA--LP	279
NP_6274424-hydroxyphenylpyruvic acid dioxygenase [Streptomyces coelicolor A3(2)]	205	AMDSIVVRNAGGGITFTLIEPDDTRVPGQIDQFLSAHDGPGVQHLAFLVDDIVGSVRSLGDRGVAFLRTPGAYYDL--LT	282
YP_0031140044-hydroxyphenylpyruvate dioxygenase [Catenulispora acidiphila DSM 44928]	218	AMDSIVVRNAGGGITFTLIEPDDTRVPGQIDQFLSAHDGPGVQHLAFLVDDIVGSVRSLGDRGVAFLRTPGAYYDL--LT	295
ABD659454-hydroxyphenylpyruvate dioxygenase [Streptomyces fungicidicus]	201	GMYSQVVQSPSGGATFTIIQPDMSRDSGQIDDFLAWHGGAGVQHLALSTDDIVATVKSFAANGAGFAQTPGAYYDV--LP	278
YP_0011593144-hydroxyphenylpyruvate dioxygenase [Salinispora tropica CNB-440]	200	AMDSKVVQSDRGAVTFTVIEPDTTRAPGQIDAFVASHGGAGVQHVAFLTEDITTAVRTCTGRGVRFLTTPPSYYEM--LP	277
YP_0015375014-hydroxyphenylpyruvate dioxygenase [Salinispora arenicola CNS-205]	224	AMNSTVVQCPSGRVTLVLLEPDTNRRAGQIDAFLAQHSGAGVQHLGLRTNDIIEAIGAMRQRGLRFARTPAAYYDD--LE	301
ZP_046041114-hydroxyphenylpyruvate dioxygenase [Micromonospora sp. ATCC 39149]	202	AMNSTVVQSRSGRVTLVLLEPDTTRRAGQIDAFLTQHAGAGVQHLGLRTDDIVEAVTALRQRGVGFARTPAAYYDD--LE	279
	99	AMNSTVVQSPSGRVTLVLLEPDRSHRPGQIDAFLTEHAGAGVQHLGLRTDDIVTAVGALRERGVRFAGTPGAYYDD--LE	176

+-+

x+x

+* +

Sequence	Start	Alignment	End
56248	276	ERIT---L-----QTHSLDDLRATNVLADEDHGGQLFQIFTASTHPRHTIFFEVIERQGAGTFGSSNIKALYEAVELERT	347
2R5V_AChain A, Hydroxymandelate Synthase Crystal Structure	276	ERIT---L-----QTHSLDDLRATNVLADEDHGGQLFQIFTASTHPRHTIFFEVIERQGAGTFGSSNIKALYEAVELERT	347
ACJ60972VEG30 [uncultured soil bacterium]	276	ERIE---L-----ATHSLDDLRATNVLADEDHGGQLFQIFTASTHPRHTIFFEVIERQGAGTFGSSNIKALYEAVELERT	347
CAC48371putative hydroxyphenyl pyruvate dioxygenase [Amycolatopsis balhimycina]	275	ERIT---L-----ETHTLDDLRSTNVLADEDHGGQLFQIFAASTHPRHTIFFEIIERQGAGTFGSSNIKALYEAVELERT	346
AAM80551HmaS [Streptomyces toyocaensis]	291	ERIA---L-----SESRVDDLRATNVLVDEDHGGQLFQIFTASTHLRHTLFFEVIERQGAETFGSANIKALYEAVELERT	362
CAD91197putative hydroxymandelate synthase [Nonomuraea sp. ATCC 39727]	281	SRIQ---I-----RGHTVDQLRATGLLADEDHGGQLFQIFTASTHPRETLFFEVIERQGARTFGGANIKALYEAVEVARS	352
AAK81835hydroxyphenyl pyruvate dioxygenase [Streptomyces lavendulae]	261	GRIE---L-----ATHSIGTLRETQLLADEDHGGQLFQIFTASTHPKRTLFFEIIERQGAGTFGSANIKALYEAVELEKA	332
ZP_046968484-hydroxyphenylpyruvate dioxygenase [Streptomyces roseosporus NRRL 15998]	282	DRLR---L-----VRHSTDALAALDILADDDHAGQLFQIFTRSVHPRRTLFFEVIERAGATTFGSGNIKALYEAVEAEQL	353
YP_0033424144-hydroxyphenylpyruvate dioxygenase [Streptosporangium roseum DSM 43021]	285	ERME---L-----GRYSVDELRRLNILVDEDHDGQLYQIFARSVHPRNTFFLELIERLGARSFGSGNISALYQAVELQQS	356
CAG15040HmaS protein [Actinoplanes teichomyceticus]	277	DRLQ---L-----TRHSVTELSRLNVLADEDHDGQLYQIFTKSTHPRGTLFFEIIERVGARTFGSGNIKALYEAVELDQA	348
CAE53378HmaS protein [Actinoplanes teichomyceticus]	275	DRLQ---L-----TRHSVTELSRLNVLADEDHDGQLYQIFTKSTHPRGTLFFEIIERVGARTFGSGNIKALYEAVELDQA	346
YP_0031022694-hydroxyphenylpyruvate dioxygenase [Actinosynnema mirum DSM 43827]	270	DRLG---P-----TRYSTAELAELNLLVDEDQDGKLYQIFARSTHPRGTFFFEIIERAGAHTFGSGNIKALYEAVEAERH	341
YP_0011595494-hydroxyphenylpyruvate dioxygenase [Salinispora tropica CNB-440]	285	ARMD---L-----ARYTVDELRSRSILVDSDHDGQLYQIFTKSVHPRNTIFLEIIERLGARGFGSGNIRALYEAVERTRE	356
YP_0033838684-hydroxyphenylpyruvate dioxygenase [Kribbella flavida DSM 17836]	282	ERVG---E-----VETPIEDLQKLGILVDRDHWGQLLQIFTESMHVRRTLFLEIIERRGAMTFGSGNIKALYEAKEREL-	352
ZP_064002134-hydroxyphenylpyruvate dioxygenase [Micromonospora sp. L5]	280	QRVG---R-----LDAPVDRLRELSILVDSDHDGQLLQIFTESMHVRRTLFLELIERRGARTFGSGNIKALYEAKERELA	351
ZP_062154194-hydroxyphenylpyruvate dioxygenase [Micromonospora aurantiaca ATCC 27029]	280	QRVG---R-----LDAPVDRLRELSILVDSDHDGQLLQIFTESMHVRRTLFLELIERRGARTFGSGNIKALYEAKERELA	351
YP_4815504-hydroxyphenylpyruvate dioxygenase [Frankia sp. CcI3]	280	ARLG---P-----VGVPVETLRELNILADRDHGGVMLQIFTASRHPRRTFFHELIDRRGAHTFGSNNIKALYEAVERQRA	351
ZP_055255754-hydroxyphenylpyruvic acid dioxygenase [Streptomyces lividans TK24]	283	ERVG---A-----MADAIEDLRETNVLADRDEWGYLLQIFTRSPYPRGTLFYEYIQRNGARGFGSSNIKALYEAVERERE	354
NP_6274424-hydroxyphenylpyruvic acid dioxygenase [Streptomyces coelicolor A3(2)]	296	ERVG---A-----MADAIEDLRETNVLADRDEWGYLLQIFTRSPYPRGTLFYEYIQRNGARGFGSSNIKALYEAVERERE	367
YP_0031140044-hydroxyphenylpyruvate dioxygenase [Catenulispora acidiphila DSM 44928]	279	ERIG--------ATDLPVDQLRPLGILVDRDHWGQMYQIFSKSMHIRRTFFWELIERHGAKTFGTSNIPALYAAKERELA	350
ABD659454-hydroxyphenylpyruvate dioxygenase [Streptomyces fungicidicus]	278	GRLG---P-----VGVPVEELSALNILADRDPSGIMLQIFTESTHPRRTLFWELIDRRGAQTFGSNNIQALYEAVERQQA	349
YP_0011593144-hydroxyphenylpyruvate dioxygenase [Salinispora tropica CNB-440]	302	TRVG---R-----VDGSVDQLREFGVLVDRDHDGQLLQIFTESMHVRRTLFLELIERRGAQTFGSGNIKALYEAKERELA	373
YP_0015375014-hydroxyphenylpyruvate dioxygenase [Salinispora arenicola CNS-205]	280	TRVG---R-----VDGSLDRLRELGVLVDRDHDGQLLQIFTESMHVRRTLFLELIERRGARTFGSGNIKALYEAKERELA	351
ZP_046041114-hydroxyphenylpyruvate dioxygenase [Micromonospora sp. ATCC 39149]	177	RRVG---P-----VDAPLDRLRELSILVDRDHGGQLLQIFTESMHVRRTLFLELIERRGALTFGSGNIKALYEAKERELA	248
		+ + * ++ +x ++	

<u>legend:</u>

* metal binding

x conserved among HmS

+ conserved among HmS and HppD

– pointing outside the active site, backbone lines binding pocket
