# Supplementary material for: Chiral Hydroxylation at the Mononuclear Nonheme Fe(II) Center of 4-(S) Hydroxymandelate Synthase – A Structure-Activity Relationship Analysis
Source: PLoS One. 2013 Jul 23;8(7):e68932. doi: 10.1371/journal.pone.0068932 (PMC3720870; doi:10.1371/journal.pone.0068932)
Supplement: Table S1 — Biochemical characteristics of C- and N-terminally tagged S. coelicolor Hms. Specific activities were determined with HPP (5 mM) as a substrate in air saturated buffer (Tris 20 mM, pH 7.5, 25°C). Parameters were determined as outlined in the Material and Methods section. (DOCX) [file pone.0068932.s008.docx]

Table S1: Biochemical characteristics of C- and N-terminally tagged *S. coelicolor* Hms.

| *Characteristic* | *C-terminal tag* | *N-terminal tag* |
| --- | --- | --- |
| Iron content after purification (%) | 30-50 | <5 |
| Theoretical molecular weight (Da) | 41,412 | 42,860 |
| Specific activity (s^-1^) | 4.5 | <10^-4^ |
| Quaternary structure | monomer | heterogeneous |
| Theoretical extinction coefficient (mL · mg^-1^ · cm^-1^) | 1.17 | 1.26 |

Specific activities were determined with HPP (5mM) as a substrate in air saturated buffer (Tris 20 mM, pH 7.5, 25°C). Parameters were determined as outlined in the Material and Methods section.

.
