# Supplementary material for: Chiral Hydroxylation at the Mononuclear Nonheme Fe(II) Center of 4-(S) Hydroxymandelate Synthase – A Structure-Activity Relationship Analysis
Source: PLoS One. 2013 Jul 23;8(7):e68932. doi: 10.1371/journal.pone.0068932 (PMC3720870; doi:10.1371/journal.pone.0068932)
Supplement: Table S2 — Parameters employed for QSAR analysis. σ is the sigma parameter as defined by Hammett; σp •, σp +, σp − are sigma para values derived from models that have radical character or positive or negative charge during the respective reaction’s transition state; σ* quantitatively describes aliphatic inductive effects [33]. The frontier energy of each monoanionic ligand’s highest occupied molecular orbital εHOMO and the atomic volume (MV) of the substituent that is fused to the α-keto-pyruvate core structure were both obtained from DFT calculations using the software Spartan (Wavefunction Inc.). * n.a. indicates that no parameter was available. (DOCX) [file pone.0068932.s009.docx]

Table S2: Parameters employed for QSAR analysis.

| *Substrate* | *Parameter* | | | | | | |
| --- | --- | --- | --- | --- | --- | --- | --- |
|  | σ | σ_p_^•^ | σ_p_^+^ | σ_p_^-^ | σ_p_^*^ | MV | ε_HOMO_ |
|  | (-) | (-) | (-) | (-) | (-) | (Å^3^) | (e. V.) |
| PP | 0 | 0.00 | 0.00 | 0.00 | 0.75 | 106 | -4.55 |
| *p*-Hydroxy-PP | -0.37 | 0.17 | -0.92 | -0.37 | n.a. | 117 | -4.60 |
| *p*-Methoxy-PP | -0.27 | 0.11 | -0.78 | -0.26 | 0.6 | 138 | -4.58 |
| *p*-Methyl-PP | -0.17 | 0.03 | -0.31 | -0.17 | 0.59 | 126 | -4.56 |
| *p*-Fluoro-PP | 0.06 | -0.07 | -0.07 | -0.03 | 0.81 | 113 | -4.66 |
| *p*-Nitro-PP | 0.78 | 0.41 | 0.79 | 1.27 | 1.26 | 137 | -5.10 |
| *p*-2-Oxo-4-phenylbutanoate | n.a.* | n.a.* | n.a.* | n.a.* | 0.22 | 126 | -4.66 |

σ is the sigma parameter as defined by Hammett; σ_p_^•^, σ_p_^+^, σ_p_^-^ are sigma para values derived from models that have radical character or positive or negative charge during the respective reaction’s transition state; σ^*^ quantitatively describes aliphatic inductive effects [33]. The frontier energy of each monoanionic ligand’s highest occupied molecular orbital ε_HOMO_ and the atomic volume (MV) of the substituent that is fused to the α-keto-pyruvate core structure were both obtained from DFT calculations using the software Spartan (Wavefunction Inc.).

* n.a. indicates that no parameter was available.
