# Supplementary material for: Chiral Hydroxylation at the Mononuclear Nonheme Fe(II) Center of 4-(S) Hydroxymandelate Synthase – A Structure-Activity Relationship Analysis
Source: PLoS One. 2013 Jul 23;8(7):e68932. doi: 10.1371/journal.pone.0068932 (PMC3720870; doi:10.1371/journal.pone.0068932)
Supplement: Table S3 — Theoretical binding energies of monoanionic aliphatic substrates from in silico docking experiments. (DOCX) [file pone.0068932.s010.docx]

Table S3: Theoretical binding energies of monoanionic aliphatic substrates from in silico docking experiments.

| *Substrate* | *Calculated binding energy* |
| --- | --- |
|  | (kcal mol^-1^) |
| HPP | -6.4 |
| 2-Oxo-4-methylthiobutyric acid | -4.3 |
| 3-Methyl-2-oxobutyric acid | -5.1 |
| 2-Oxobutyric acid | -4.3 |
| 2-Oxooctanoic acid | -5.9 |
| 2-Oxoglutaric acid | -5.3 |
| 4-Methyl-2-oxopentanoic acid | -5.2 |
| 2-Oxovaleric acid | -4.8 |
