# Supplementary material for: Chiral Hydroxylation at the Mononuclear Nonheme Fe(II) Center of 4-(S) Hydroxymandelate Synthase – A Structure-Activity Relationship Analysis
Source: PLoS One. 2013 Jul 23;8(7):e68932. doi: 10.1371/journal.pone.0068932 (PMC3720870; doi:10.1371/journal.pone.0068932)
Supplement: Table S4 — Theoretical substrate and product binding energies of monoanionic aromatic ligands from in silico docking experiments. (DOCX) [file pone.0068932.s011.docx]

Table S4: Theoretical substrate and product binding energies of monoanionic aromatic ligands from in silico docking experiments.

|  | *Calculated binding energy* (kcal mol^-1^) | | |
| --- | --- | --- | --- |
| *Primary substrate* | *Substrate* | *(S)-Product* | *(R)-Product* |
| Phenylpyruvate | -6.4 | -7.5 | -7.1 |
| *p*-Hydroxy-PP | -6.4 | -7.7 | -7.1 |
| *p*-Fluoro-PP | -6.6 | -7.8 | -7.1 |
| *p*-Methyl-PP | -6.7 | -7.9 | -7.1 |
| *p*-Methoxy-PP | -6.3 | -7.5 | -6.9 |
| *p*-2-Oxo-4-phenylbutanoate | -5.7 | -6.4 | -6.3 |
