# Supplementary material for: Chiral Hydroxylation at the Mononuclear Nonheme Fe(II) Center of 4-(S) Hydroxymandelate Synthase – A Structure-Activity Relationship Analysis
Source: PLoS One. 2013 Jul 23;8(7):e68932. doi: 10.1371/journal.pone.0068932 (PMC3720870; doi:10.1371/journal.pone.0068932)
Supplement: Table S5 — Distances of aromatic ligand substituents to residues in the S. coelicolor Hms active site pocket derived from in silico docking studies. Distances (d) to the respective closest heavy atoms are given, as indicated in Figure 4. (DOCX) [file pone.0068932.s012.docx]

Table S5: Distances of aromatic ligand substituents to residues in the *S. coelicolor* Hms active site pocket derived from in silico docking studies.

| *Primary substrate* | *4-Hydroxy-PP* | *PP* | *4-Methoxy-PP* | *2-Oxo-4-phenylbutanoate* |
| --- | --- | --- | --- | --- |
|  | d (Å) | d (Å) | d (Å) | d (Å) |
| *Substrate-HMS complexes* |  |  |  |  |
| C_β_-Glu210 | 4.08 | 4.63 | 3.08 | 4.51 |
| N-Glu210 | 4.71 | 5.51 | 3.62 | 5.25 |
| N-Ser209 | 4.40 | 5.64 | 3.59 | 5.01 |
| O_G_-Ser208 | 3.14 | 4.18 | 3.61 | 3.65 |
| C_G2_-Val223 | 3.63 | 3.37 | 3.28 | 3.49 |
| *Product-HMS complexes* |  |  |  |  |
| C_β_-Glu210 | 3.83 | 4.76 | 3.65 | 3.78 |
| N-Glu210 | 4.41 | 5.68 | 4.21 | 4.67 |
| N-Ser209 | 4.98 | 6.24 | 4.23 | 5.53 |
| O_G_-Ser208 | 4.58 | 5.38 | 3.26 | 5.02 |
| C_G2_-Val223 | 3.54 | 3.76 | 3.62 | 4.01 |

Distances (d) to the respective closest heavy atoms are given, as indicated in Figure 4.
