# Supplementary material for: Chiral Hydroxylation at the Mononuclear Nonheme Fe(II) Center of 4-(S) Hydroxymandelate Synthase – A Structure-Activity Relationship Analysis
Source: PLoS One. 2013 Jul 23;8(7):e68932. doi: 10.1371/journal.pone.0068932 (PMC3720870; doi:10.1371/journal.pone.0068932)
Supplement: Protocol S1 — (DOC) [file pone.0068932.s013.doc]

**Protocol S1** - Chiral Hydroxylation at the Mononuclear Nonheme Fe(II) Center of 4-(S) Hydroxymandelate Synthase – a Structure-Activity Relationship Analysis

*Cristiana M. L. Di Giur*o*, Cornelia Konstantinovics, Uwe Rinner, Christina Nowikow, Erich Leitner and Grit D. Straganz*

**Experimental procedure for the preparation of α-oxo acids VII-IX**

All non-aqueous reactions were carried out under a positive pressure of argon using oven-dried (100 °C) or flame-dried glassware (under vacuum) unless otherwise noted.

Dimethyl sulfoxide was dried by distillation from calcium hydride under reduced pressure. Dichloromethane was purified by distillation prior to use. Dry solvents were stored under an argon atmosphere over a molecular sieve (4Å).

All other commercially available reagents were used without further purification. Except if indicated otherwise, reactions were magnetically stirred and monitored by thin layer chromatography using Merck silica gel 60-F254 glass plates. The plates were developed with a mixture of hexane/ethyl acetate or toluene/ethyl acetate. Unless the compound was colored, UV-active spots were detected at longwave UV (254 nm) or shortwave (180 nm). Most plates were additionally treated with one of the following visualization reagents: CAM [H_2_SO_4_ (conc., 22 mL), phosphormolybdic acid (20 g), Ce(SO_4_)_2_ (0.5 g), 378 mL H_2_O)] or silica gel impregnated with iodine.

Preparative column chromatography and flash chromatography was performed with silica gel 60 from Merck (0.040-0.063 µm, 240-400 mesh).

Concentration under reduced pressure was performed by rotary evaporation at 30 °C at the appropriate pressure, unless otherwise stated. Yields refer to chromatographically purified and spectroscopically pure compounds, unless otherwise stated.

NMR spectra were recorded either on a Bruker Avance AV 400, DRX 400, or DRX 600 MHz spectrometer. Unless otherwise stated, all NMR spectra were measured in CDCl_3_ solutions and referenced to the residual CDCl_3_ signal (^1^H, δ = 7.26, ^13^C, δ = 77.16). All ^1^H and ^13^C shifts are given in ppm (s = singlet, d = doublet, dd = doublet of doublets, t = triplet, q = quartet, m = multiplet, br = broadened signal). Coupling constants *J* are given in Hz.

**4-Methoxybenzaldehyde I:**

A suspension of 4.5 g (4 eq., 81.8 mmol) KOH in 104 mL of DMSO was stirred for 20 min before 4-hydroxybenzaldehyde (2.5 g, 20.45 mmol) and iodomethane (2 eq, 2.55 ml) were added. Stirring at room temperature was continued until TLC (PE:EE 2:1) showed no remaining starting material. The orange solution was diluted with dichloromethane (300 mL) and poured into ice-water. The aqueous phase was extracted with dichloromethane and the combined organic extracts were washed with water, dried over Na_2_SO_4_ and evaporated to yield 2.7 g of crude 4-methoxybenzaldehyde (quant.), which was used without further purification. ^1^H-NMR (400MHz, CDCl_3_), δ [ppm] 9.88 (s, 1H); 7.83 (d, 2H, 8.86 Hz); 6.99 (d, 2H, 8.86 Hz); 3.88 (s, 3H).

**5-(4-Methoxybenzylidene)imidazolidine-2,4-dione IV:**

A mixture of ammonium acetate (1.6 g; 1.0 eq), hydantoin (2.0 g; 1.0 eq) and 2.7 g **I** (20.4 mmol) was heated in 18 mL acetic acid under reflux for 5 hours (120 °C). The hot solution was poured into 20 mL of cold water and warmed to room temperature. The yellow precipitate was filtered and washed with small portions of cold water and hexanes. Subsequent drying afforded 3.5 g (16.3 mmol, 80%) of hydantoin **IV**. ^1^H-NMR (400MHz, DMSO), δ [ppm] 10.75 (bs, 2H); 7.58 (d, 2H, 8.83 Hz); 6.95 (d, 2H, 8.83Hz); 6.38 (s, 1H); 3.79 (s, 3H). ^13^C-NMR (400MHz, DMSO), δ [ppm] 165.63; 159.44; 155.66; 126.11; 125.45 (^13^C_q_); 131.08, 114.30 (2x ^13^CH); 108.64(^13^CH); 55.27 (^13^CH_3_).

**5-(4-Methylbenzylidene)imidazolidine-2,4-dione V:**

Analogously to the preparation of 5-(4-methoxybenzylidene)imidazolidine-2,4-dione (**IV)**, 1.0 g (8.3 mmol) of tolualdehyde **II**, 840 mg of hydantoin and 2.5 g of ammonium acetate afforded 1.4 g (7 mmol, 84%) of corresponding hydantoin **V**. ^1^H-NMR (400MHz, DMSO), δ [ppm] 10.83 (bs, 2H); 7.52 (d, 2H, 8.25 Hz); 7.22 (d, 2H, 8.25 Hz); 6.39 (s, 1H); 2.33 (s, 3H). ^13^C-NMR (400MHz, DMSO), δ[ppm] 165.59, 155.65, 138.14, 130.12, 127.21 (^13^C_q_); 129.39, 129.36 (2x ^13^CH); 108.49 (^13^CH); 20.92 (^13^CH_3_).

**5-(4-Fluorobenzylidene)imidazolidine-2,4-dione III:**

Analogously to the preparation of 5-(4-methoxybenzylidene)imidazolidine-2,4-dione **IV**, 2.0 g (16.1 mmol) of 4-fluorobenzaldehyde **III**, 1.6 g hydantoin and 5.0 g of ammonium acetate yielded 2.6 g of compound **VI** (12.6 mmol, 79%).  ^1^H-NMR (400MHz, DMSO), δ [ppm] 7.63-7.71 (m, 2H); 7.19-7.28 (m, 2H); 6.41 (s, 1H). ^13^C-NMR (400MHz, DMSO), δ [ppm] 162.99, 160.53 (^13^C-F); 165.71, 155.95, 129.66, 127.98 (^13^C_q_); 131.56, 131.48, 115.81, 115.60, 107.01 (^13^CH). ^19^F-NMR (600MHz, DMSO), δ [ppm] -112.74.

**Sodium 3-(4-methoxyphenyl)-2-oxopropanoate VII:**

A three necked flask equipped with reflux condenser and septum was charged with 1.5 g of hydantoin **IV** and set under argon atmosphere. After adding NaOH (20%, 32 mL), the apparatus was again flushed with argon. The mixture was heated under reflux for 3 hours, then cooled to 0°C and adjusted to pH 7 by slow addition of HCl_conc_. After addition of 650 mg NaHCO_3_, the mixture was extracted with diethyl ether in a liquid-liquid extraction apparatus for 3 hours. Subsequent addition of 9 mL HCl_conc_ and further extraction extracted the product into the organic phase. The solvents were removed *in vacuo*, yielding a yellow oil which was taken up in 70 mL H_2_O and readjusted to pH 7 by addition of 1 M NaOH. Freezing with liquid nitrogen and lyophilization gave white crystals of **VII** (864 mg, 4 mmol, 61%). ^1^H-NMR (400MHz, D_2_O), δ [ppm] 7.17 (d, 2H, 8.48 Hz); 6.97 (d, 2H, 8.48 Hz); 4.00 (s, 2H); 3.80 (s, 3H). ^13^C-NMR (400MHz, D_2_O), δ [ppm] 181.29, 157.72, 157.24, 130.47 (^13^C_q_); 130.70, 114.49 (2x ^13^CH); 55.77 (^13^CH_3_); 43.83 (^13^CH_2_).

**Sodium 2-oxo-3-p-tolylpropanoate VIII:**

Analogously to the preparation of **VII**, 1.4 g (6.9 mmol) of hydantoin **V** afforded 916 mg of tolylpyruvate **VIII** (4.6 mmol, 65%) as a white solid. ^1^H-NMR (400MHz, D_2_O), δ [ppm] 7.19 (d, 2H, 7.19 Hz); 7.12 (d, 2H, 7.19 Hz); 4.00 (s, 2H); 2.27 (s, 3H). ^13^C-NMR (400MHz, D_2_O), δ [ppm] 203.10, 138.11, 137.50, 130.02 (^13^C_q_); 130.16, 129.82 (2x ^13^CH); 45.78 (^13^CH_2_), 20.50 (^13^CH_3_).

**Sodium 3-(4-fluorophenyl)-2-oxopropanoate IX:**

Analogously to the preparation of **VII**, 2.0 g (9.7 mmol) of hydantoin **VI** yielded 1.22 g (6.0 mmol, 62%) of compound **IX** as a white solid. ^1^H-NMR (400MHz, D_2_O), δ [ppm] 7.23-7.33 (m, 2H); 7.13-7.22 (m, 2H); 4.13 (s, 2H). ^13^C-NMR (400MHz, D_2_O), δ [ppm] 204.32, 181.62, 162.92, 130.13 (^13^C_q_); 131.94, 131.86, 115.96, 115.75 (^13^CH); 45.40 (^13^CH_2_). ^19^F-NMR (600MHz, D_2_O), δ [ppm] -116.80.
