# Supplementary material for: Chiral Hydroxylation at the Mononuclear Nonheme Fe(II) Center of 4-(S) Hydroxymandelate Synthase – A Structure-Activity Relationship Analysis
Source: PLoS One. 2013 Jul 23;8(7):e68932. doi: 10.1371/journal.pone.0068932 (PMC3720870; doi:10.1371/journal.pone.0068932)
Supplement: Protocol S2 — (PDF) [file pone.0068932.s014.pdf]

Protocol S2 - Chiral Hydroxylation at the Mononuclear Nonheme Fe(II) Center of 4-(S) Hydroxymandelate Synthase – a Structure-Activity Relationship Analysis

Cristiana M. L. Di Giuro, Cornelia Konstantinovics, Uwe Rinner, Christina Nowikow, Erich Leitner and Grit D. Straganz

Expression and biochemical characterization of N- and C-terminally tagged variants of Hms from *S. coelicolor* A3(2) – a concise summary

The Hms gene from *Streptomyces coelicolor* A3(2) was cloned into expression vectors, whereby a streptavidin affinity tag was attached C- and N-terminally, respectively. The two Hms variants were over-expressed and purified as outlined in the Experimental Section. As the Hms variant with a cleavable N-terminal strep-tag showed structural instability and was not functional (Table S1), the Hms variant bearing the C-terminal affinity tag was used throughout further studies. The resulting protein showed >95 % purity, as estimated from the SDS gel (Figure S1). The size of the protein on the SDS gel was ~ 41 kDa under both, reducing and non-reducing conditions, which corresponds to the theoretical mass of strep-tagged Hms of 41,412 Da. Gel filtration using a Superdex size-exclusion column showed a single homogenous peak in the elution profile that corresponded to a molecular mass of ~ 45 kDa, which suggests that Hms is present in its monomeric form under the applied conditions. The secondary structure composition of Hms was assessed via CD spectroscopy. C-terminally tagged Hms showed a composition of 16% alpha helix 33% beta sheet and 20% of random coil. Properties of both, N- and C-terminally strep-tagged Hms are summarized in Table S1. The enzymatic activity of Hms towards its native substrate was determined under standard conditions (Tris/HCl buffer, pH 7.5, 20 mM, 25°C) and at air saturation. Addition of 1mM DTT to the assay or the use of HEPES buffer (20 mM, pH 7.0), which have previously been applied to assess the activity of nonheme Fe(II) dioxygenases had no significant effects on the enzymatic activity of Hms.
